# Supplementary figures and images for: Segal’s Law, 16S rRNA gene sequencing, and the perils of foodborne pathogen detection within the American Gut Project
Source: PeerJ. 2017 Jun 22;5:e3480. doi: 10.7717/peerj.3480 (PMC5483036; doi:10.7717/peerj.3480)

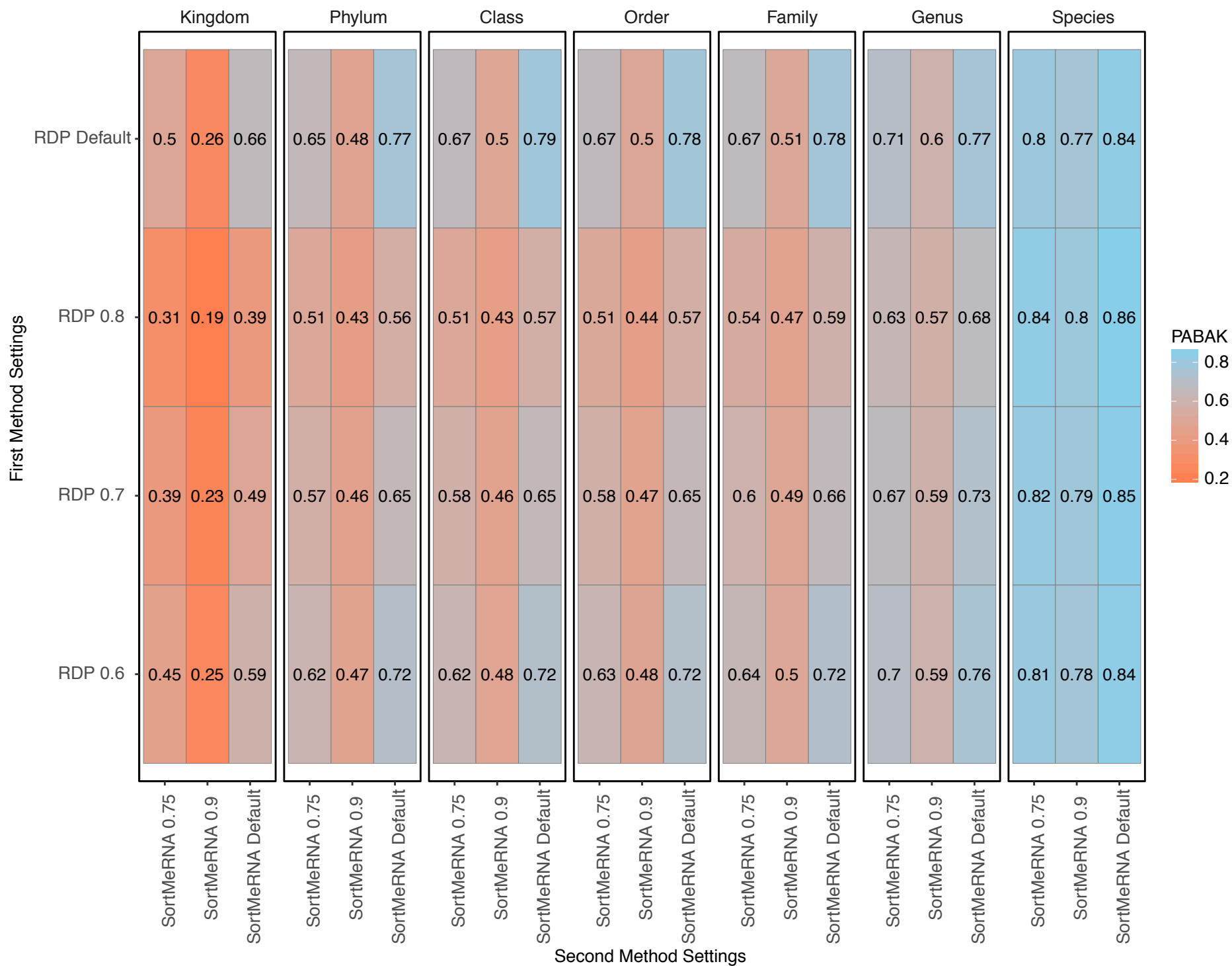

Supplement: Supplemental Information 1 — Values represent the average PABAK across samples within each comparison. PABAK incorporates a measure of how often methods may agree by chance and varies from −1 to 1 where 1 denotes complete agreement among methods (i.e., classifiers). Results are presented to not suggest methods are more incongruent at the Kingdom level than lower taxonomic levels (e.g., Species) but rather to illustrate the problems of using such an inter-rater agreement statistic like PABAK on datasets with millions of observations and only a relatively small number of categories to which they can be assigned (e.g., 16s RNA gene datasets and higher taxonomic ranks like Kingdom that have few categories). [file peerj-05-3480-s001.pdf]
