# Supplementary material for: Segal’s Law, 16S rRNA gene sequencing, and the perils of foodborne pathogen detection within the American Gut Project
Source: PeerJ. 2017 Jun 22;5:e3480. doi: 10.7717/peerj.3480 (PMC5483036; doi:10.7717/peerj.3480)
Supplement: Supplemental Information 2 [file peerj-05-3480-s002.pdf]

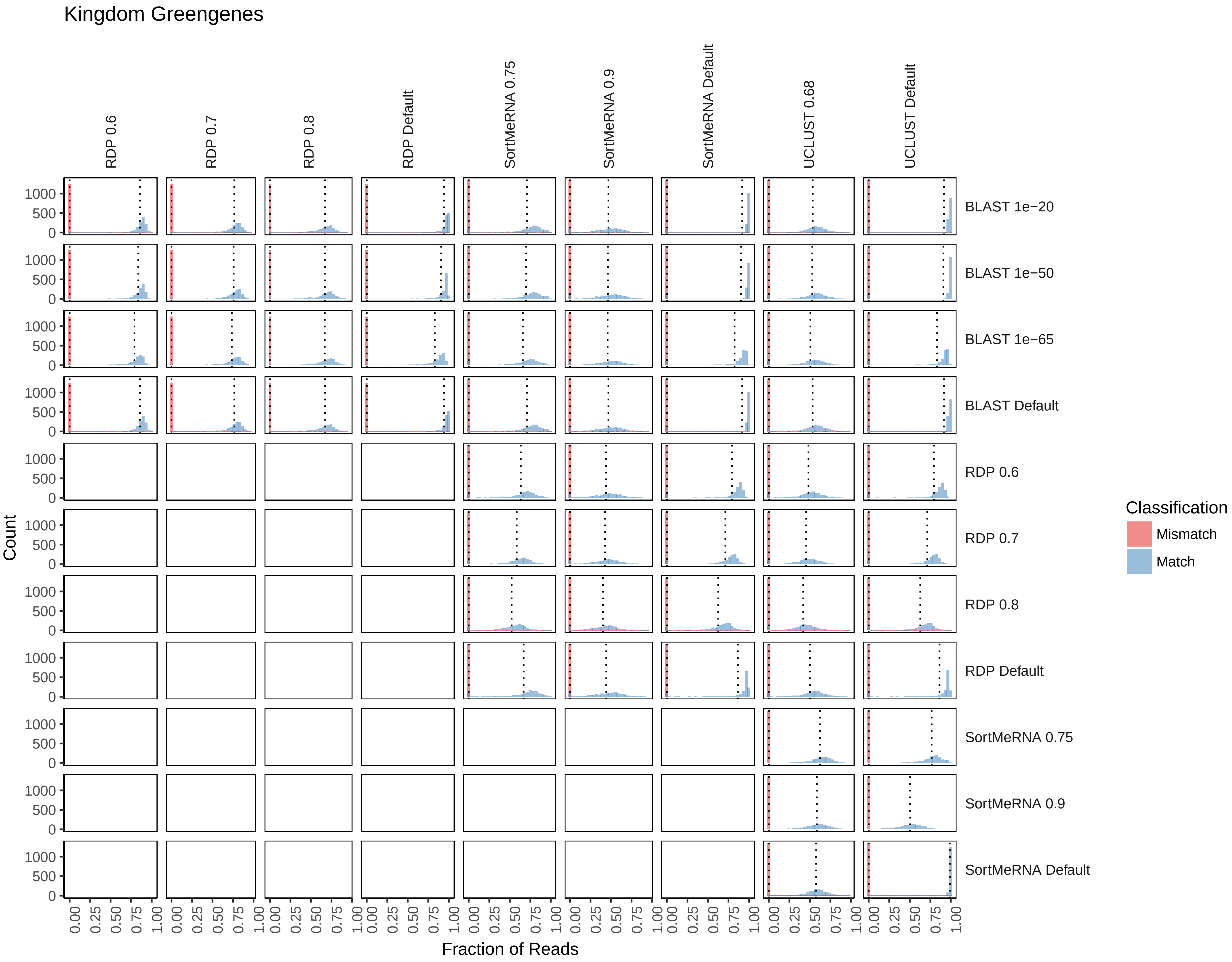

Kingdom Greengenes
RDP 0.6
RDP 0.7
RDP 0.8
RDP Default
SortMeRNA 0.75
SortMeRNA 0.9
SortMeRNA Default
UCLUST 0.68
UCLUST Default
BLAST 1e−20
BLAST 1e−50
BLAST 1e−65
BLAST Default
RDP 0.6
RDP 0.7
RDP 0.8
RDP Default
SortMeRNA 0.75
SortMeRNA 0.9
SortMeRNA Default
Count
1000
500
0
0.00
0.25
0.50
0.75
1.00
Fraction of Reads
Classification
Mismatch
Match

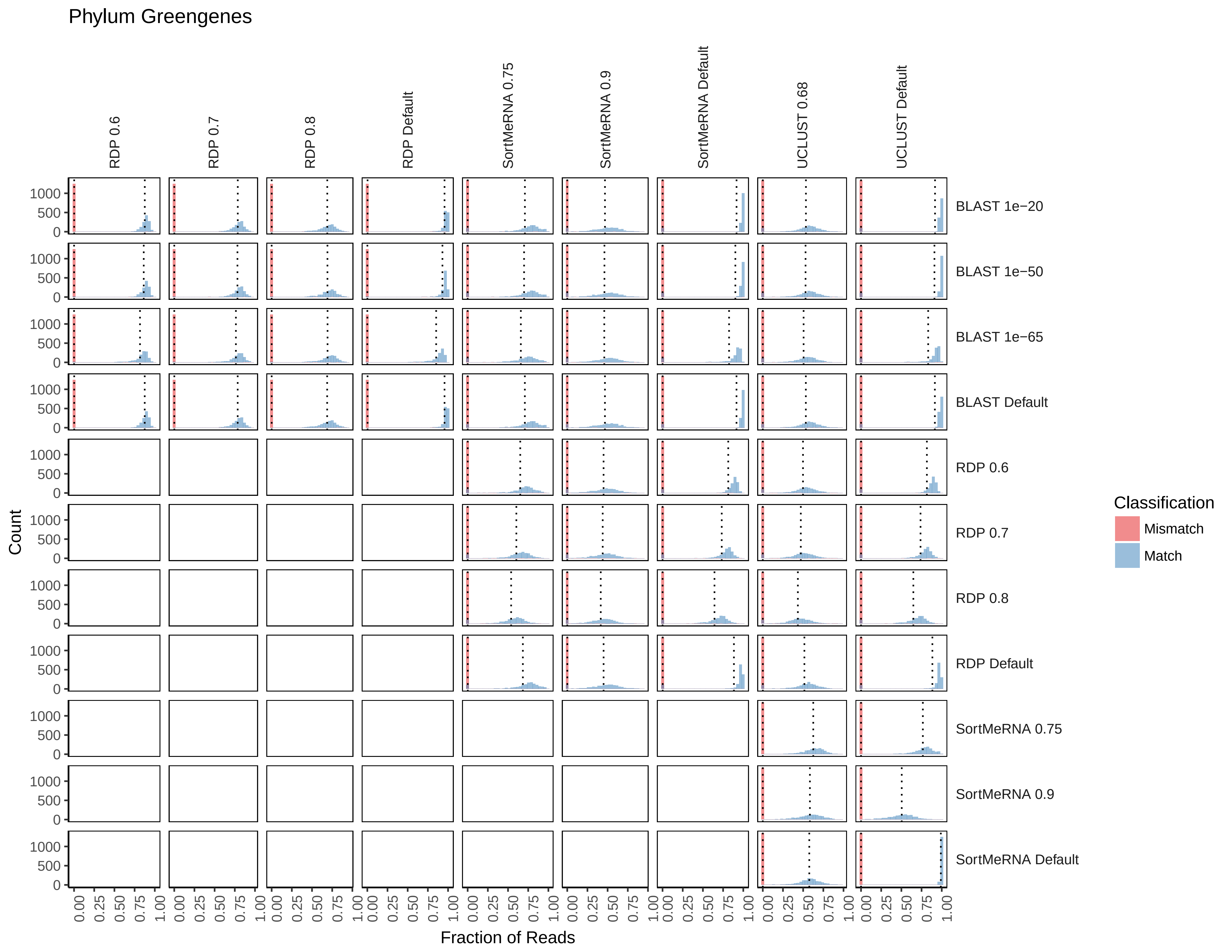
Phylum Greengenes
RDP 0.6
RDP 0.7
RDP 0.8
RDP Default
SortMeRNA 0.75
SortMeRNA 0.9
SortMeRNA Default
UCLUST 0.68
UCLUST Default
BLAST 1e−20
BLAST 1e−50
BLAST 1e−65
BLAST Default
RDP 0.6
RDP 0.7
RDP 0.8
RDP Default
SortMeRNA 0.75
SortMeRNA 0.9
SortMeRNA Default
Count
1000
500
0
0.00
0.25
0.50
0.75
1.00
Fraction of Reads
Classification
Mismatch
Match

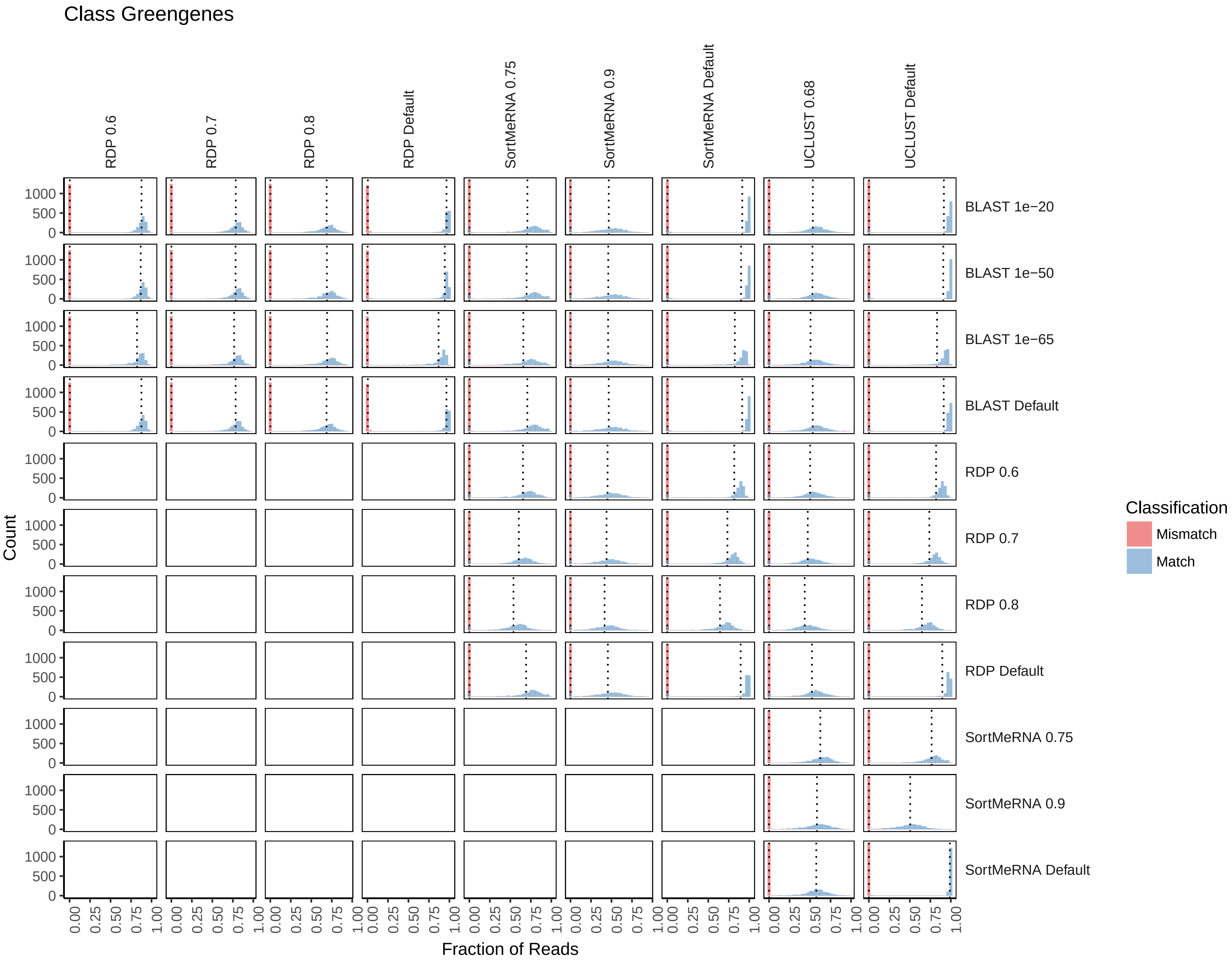
Class Greengenes
RDP 0.6
RDP 0.7
RDP 0.8
RDP Default
SortMeRNA 0.75
SortMeRNA 0.9
SortMeRNA Default
UCLUST 0.68
UCLUST Default
BLAST 1e−20
BLAST 1e−50
BLAST 1e−65
BLAST Default
RDP 0.6
RDP 0.7
RDP 0.8
RDP Default
SortMeRNA 0.75
SortMeRNA 0.9
SortMeRNA Default
Count
1000
500
0
0.00
0.25
0.50
0.75
1.00
Fraction of Reads
Classification
Mismatch
Match

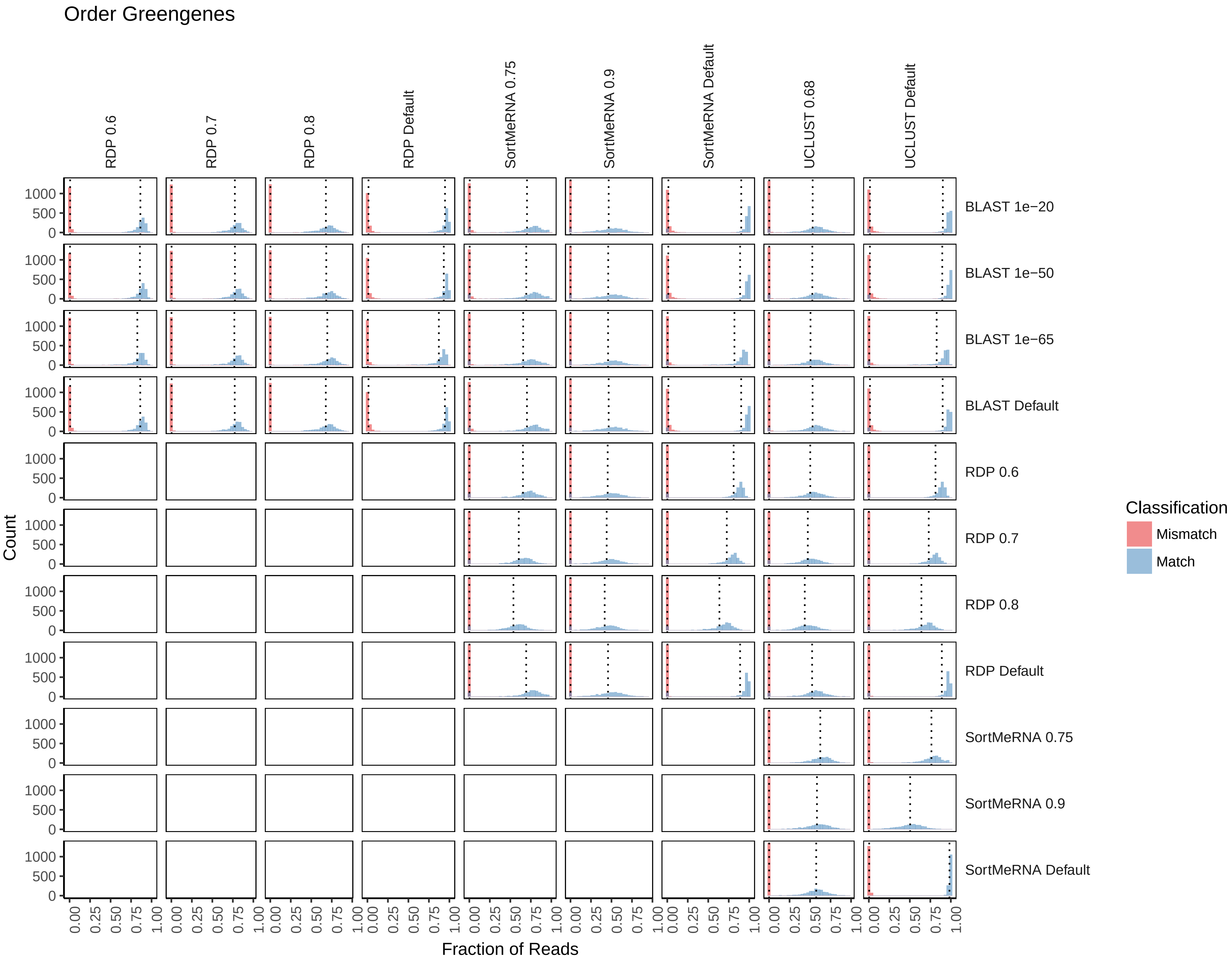

Order Greengenes
RDP 0.6
RDP 0.7
RDP 0.8
RDP Default
SortMeRNA 0.75
SortMeRNA 0.9
SortMeRNA Default
UCLUST 0.68
UCLUST Default
BLAST 1e−20
BLAST 1e−50
BLAST 1e−65
BLAST Default
RDP 0.6
RDP 0.7
RDP 0.8
RDP Default
SortMeRNA 0.75
SortMeRNA 0.9
SortMeRNA Default
Count
1000
500
0
0.00
0.25
0.50
0.75
1.00
Fraction of Reads
Classification
Mismatch
Match

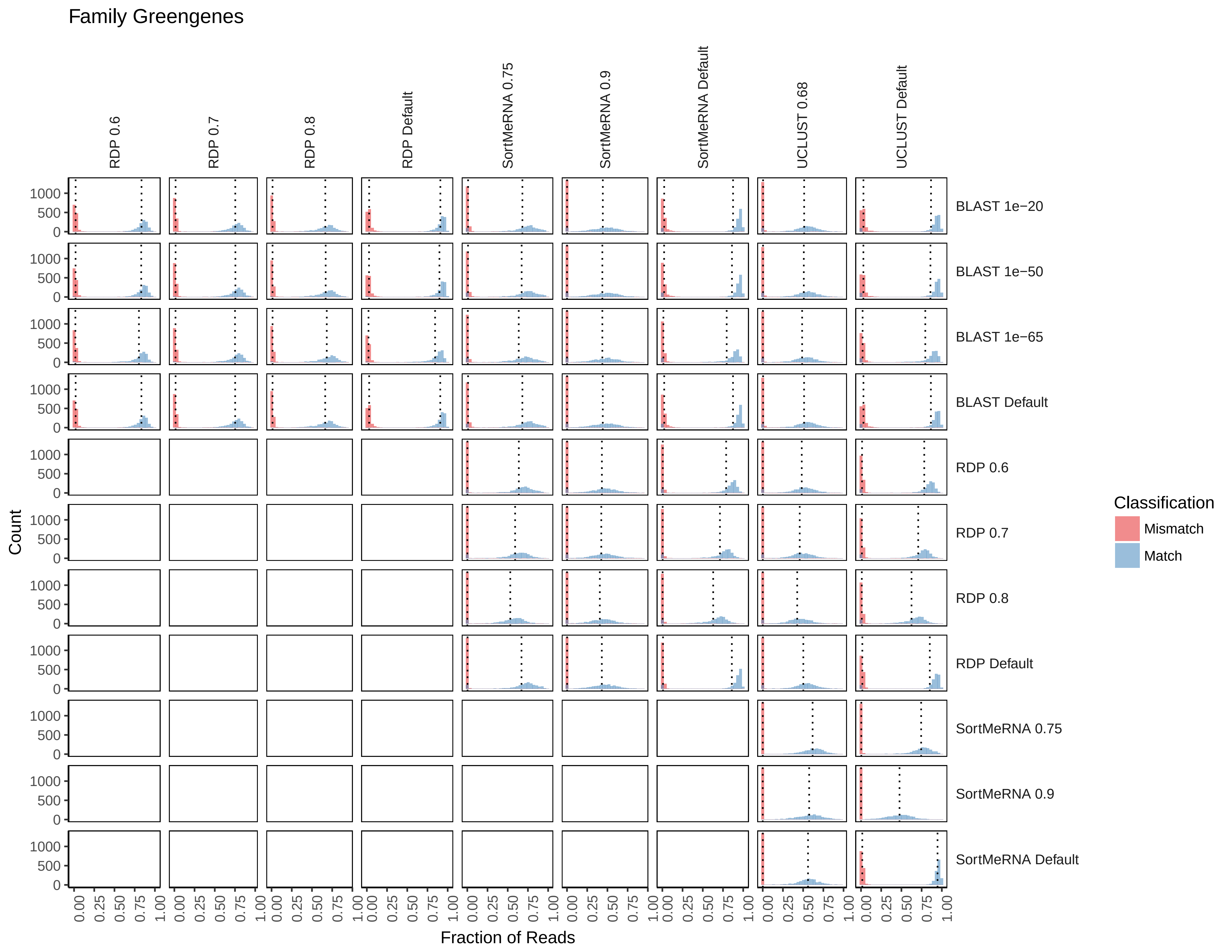

Family Greengenes
RDP 0.6
RDP 0.7
RDP 0.8
RDP Default
SortMeRNA 0.75
SortMeRNA 0.9
SortMeRNA Default
UCLUST 0.68
UCLUST Default
BLAST 1e−20
BLAST 1e−50
BLAST 1e−65
BLAST Default
RDP 0.6
RDP 0.7
RDP 0.8
RDP Default
SortMeRNA 0.75
SortMeRNA 0.9
SortMeRNA Default
Count
1000
500
0
Fraction of Reads
0.00
0.25
0.50
0.75
1.00
Classification
Mismatch
Match

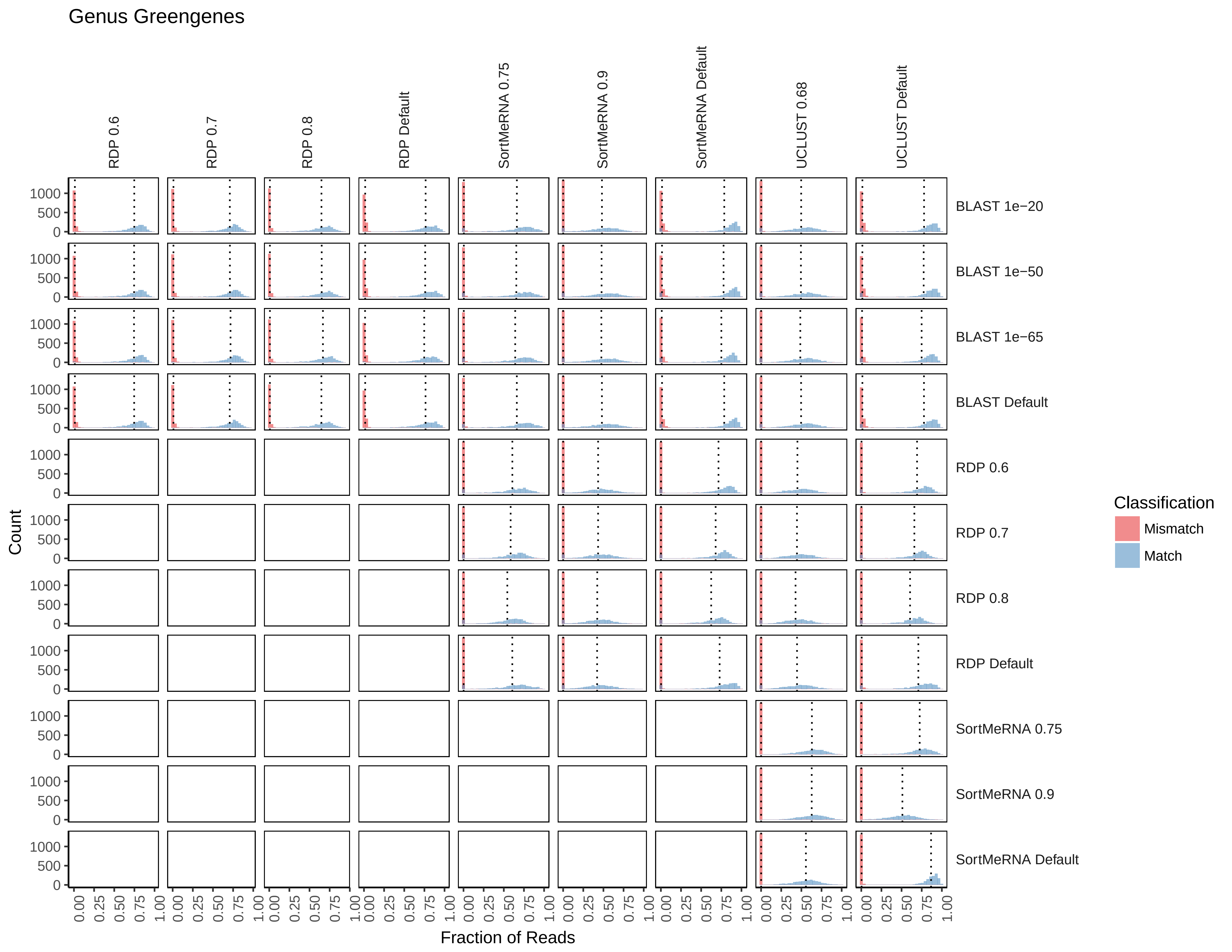

Genus Greengenes
RDP 0.6
RDP 0.7
RDP 0.8
RDP Default
SortMeRNA 0.75
SortMeRNA 0.9
SortMeRNA Default
UCLUST 0.68
UCLUST Default
BLAST 1e−20
BLAST 1e−50
BLAST 1e−65
BLAST Default
RDP 0.6
RDP 0.7
RDP 0.8
RDP Default
SortMeRNA 0.75
SortMeRNA 0.9
SortMeRNA Default
Count
1000
500
0
0.00 0.25 0.50 0.75 1.00
Fraction of Reads
Classification
Mismatch
Match

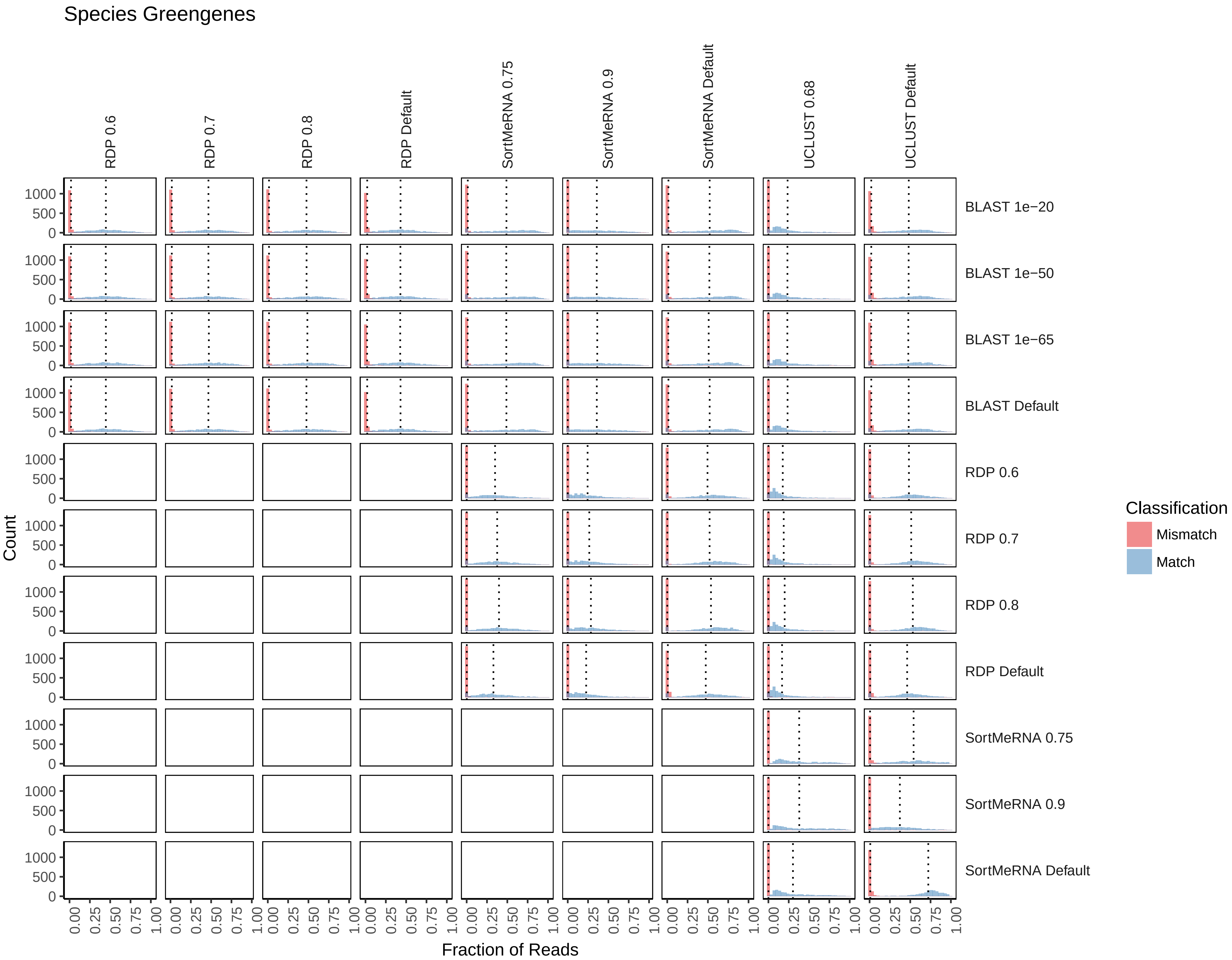

Species Greengenes
RDP 0.6
RDP 0.7
RDP 0.8
RDP Default
SortMeRNA 0.75
SortMeRNA 0.9
SortMeRNA Default
UCLUST 0.68
UCLUST Default
BLAST 1e−20
BLAST 1e−50
BLAST 1e−65
BLAST Default
RDP 0.6
RDP 0.7
RDP 0.8
RDP Default
SortMeRNA 0.75
SortMeRNA 0.9
SortMeRNA Default
Count
1000
500
0
0.00
0.25
0.50
0.75
1.00
Fraction of Reads
Classification
Mismatch
Match

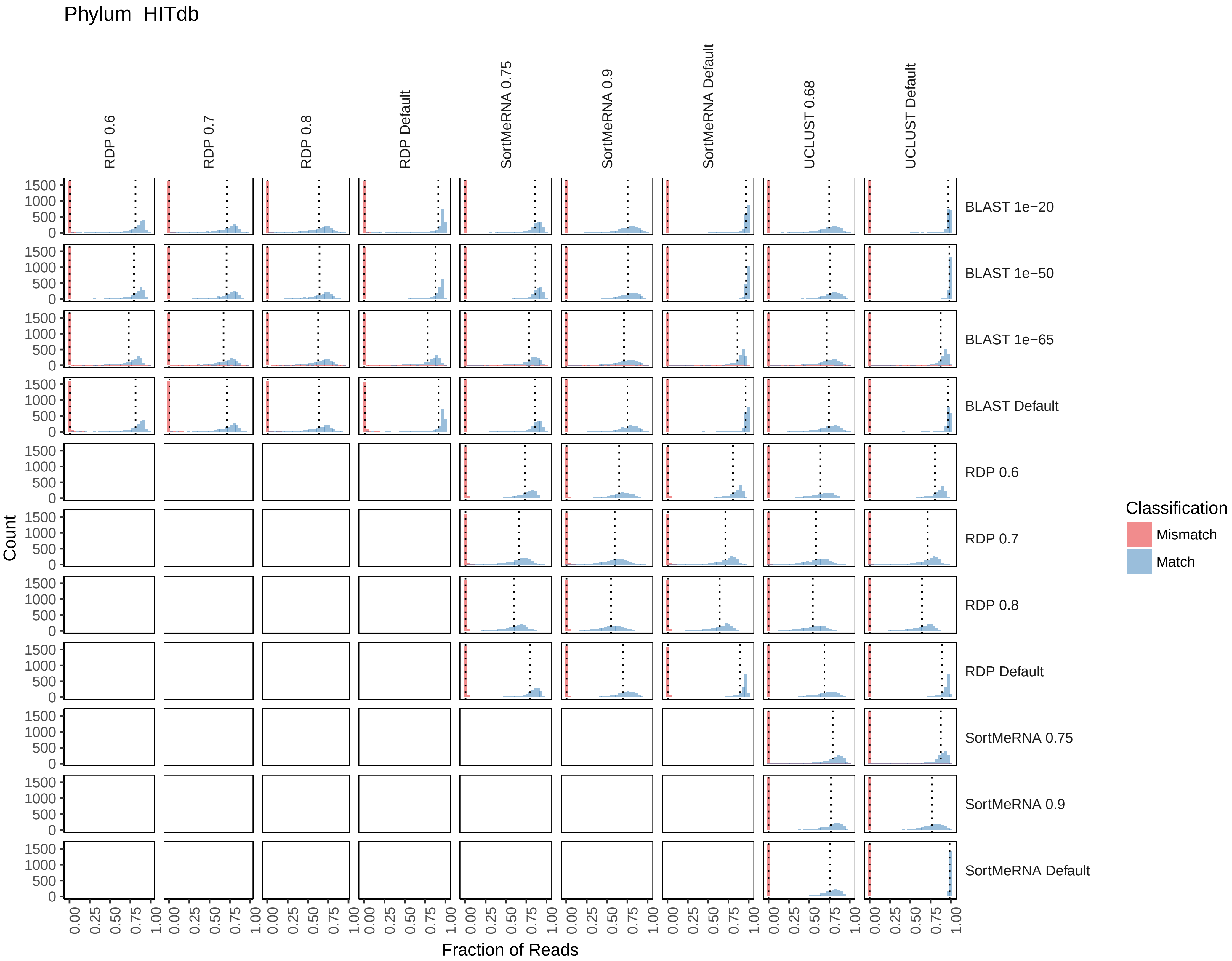

Phylum HITdb
RDP 0.6
RDP 0.7
RDP 0.8
RDP Default
SortMeRNA 0.75
SortMeRNA 0.9
SortMeRNA Default
UCLUST 0.68
UCLUST Default
BLAST 1e−20
BLAST 1e−50
BLAST 1e−65
BLAST Default
RDP 0.6
RDP 0.7
RDP 0.8
RDP Default
SortMeRNA 0.75
SortMeRNA 0.9
SortMeRNA Default
Count
0
500
1000
1500
Fraction of Reads
0.00
0.25
0.50
0.75
1.00
Classification
Mismatch
Match

Class HITdb

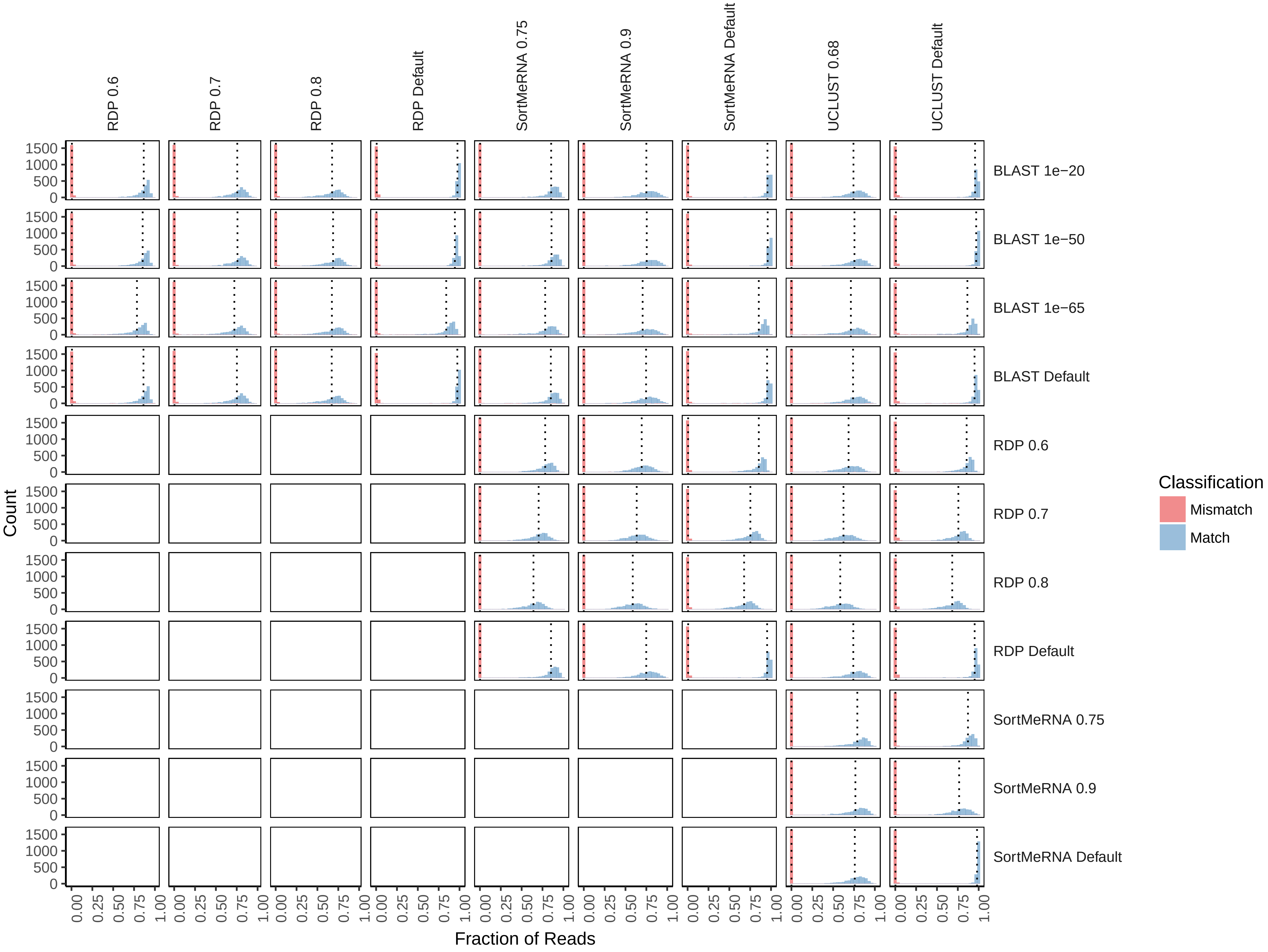

RDP 0.6
RDP 0.7
RDP 0.8
RDP Default
SortMeRNA 0.75
SortMeRNA 0.9
SortMeRNA Default
UCLUST 0.68
UCLUST Default
BLAST 1e−20
BLAST 1e−50
BLAST 1e−65
BLAST Default
RDP 0.6
RDP 0.7
RDP 0.8
RDP Default
SortMeRNA 0.75
SortMeRNA 0.9
SortMeRNA Default
Count
0
500
1000
1500
Fraction of Reads
0.00
0.25
0.50
0.75
1.00
Classification
Mismatch
Match

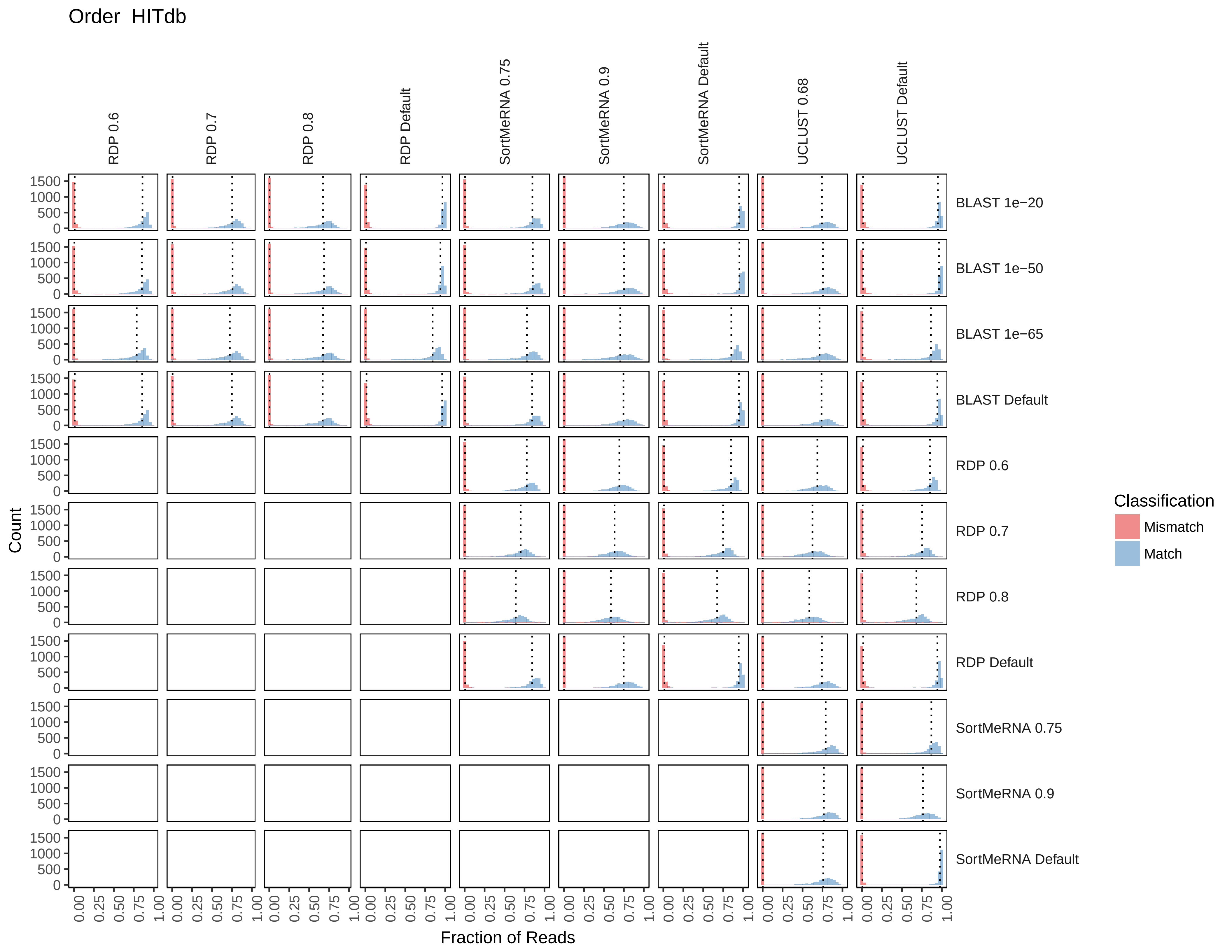

Order HITdb
RDP 0.6
RDP 0.7
RDP 0.8
RDP Default
SortMeRNA 0.75
SortMeRNA 0.9
SortMeRNA Default
UCLUST 0.68
UCLUST Default
BLAST 1e−20
BLAST 1e−50
BLAST 1e−65
BLAST Default
RDP 0.6
RDP 0.7
RDP 0.8
RDP Default
SortMeRNA 0.75
SortMeRNA 0.9
SortMeRNA Default
Count
0
500
1000
1500
0.00
0.25
0.50
0.75
1.00
Fraction of Reads
Classification
Mismatch
Match

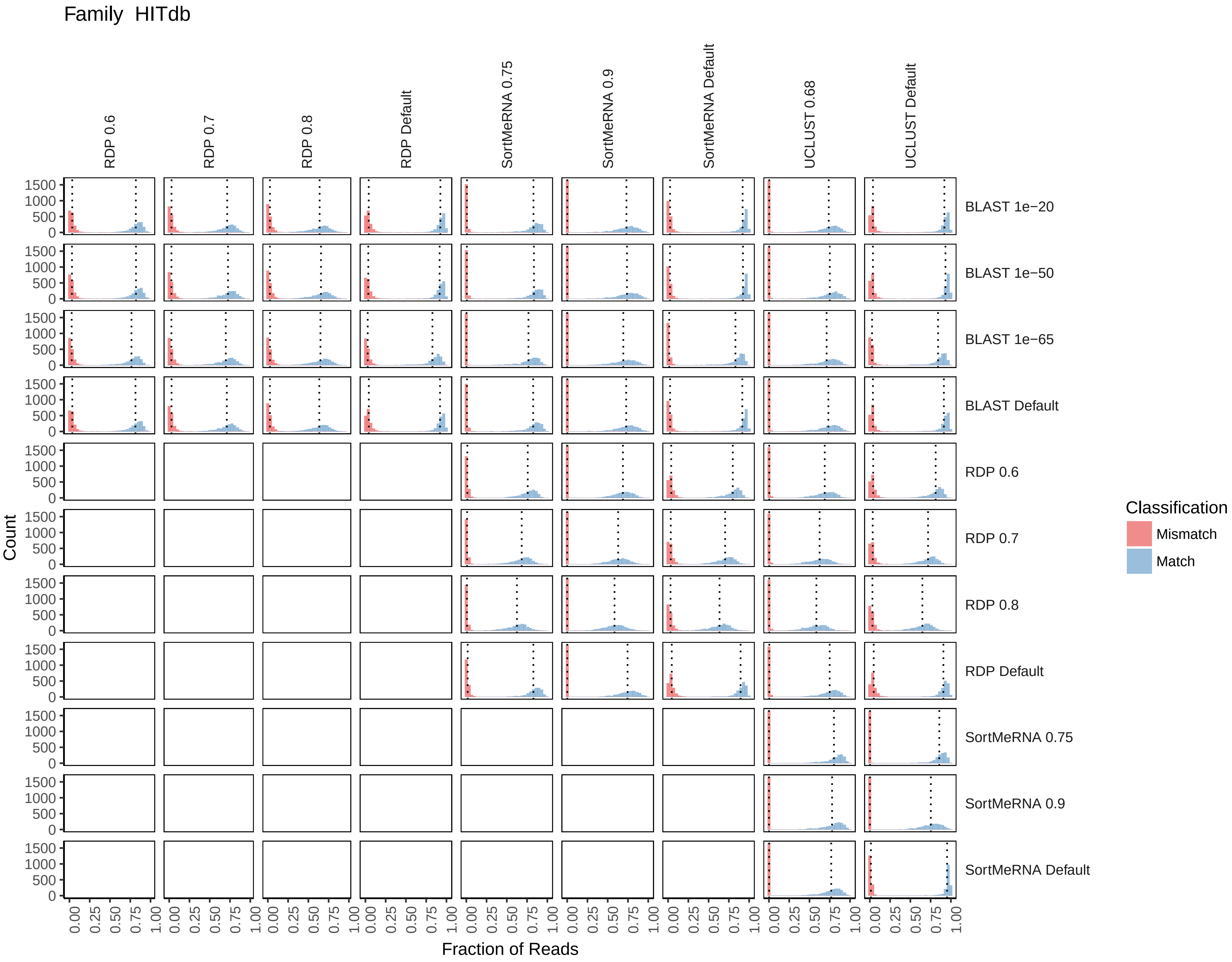

Family HITdb
RDP 0.6
RDP 0.7
RDP 0.8
RDP Default
SortMeRNA 0.75
SortMeRNA 0.9
SortMeRNA Default
UCLUST 0.68
UCLUST Default
BLAST 1e−20
BLAST 1e−50
BLAST 1e−65
BLAST Default
RDP 0.6
RDP 0.7
RDP 0.8
RDP Default
SortMeRNA 0.75
SortMeRNA 0.9
SortMeRNA Default
Count
0
500
1000
1500
0.00
0.25
0.50
0.75
1.00
Fraction of Reads
Classification
Mismatch
Match

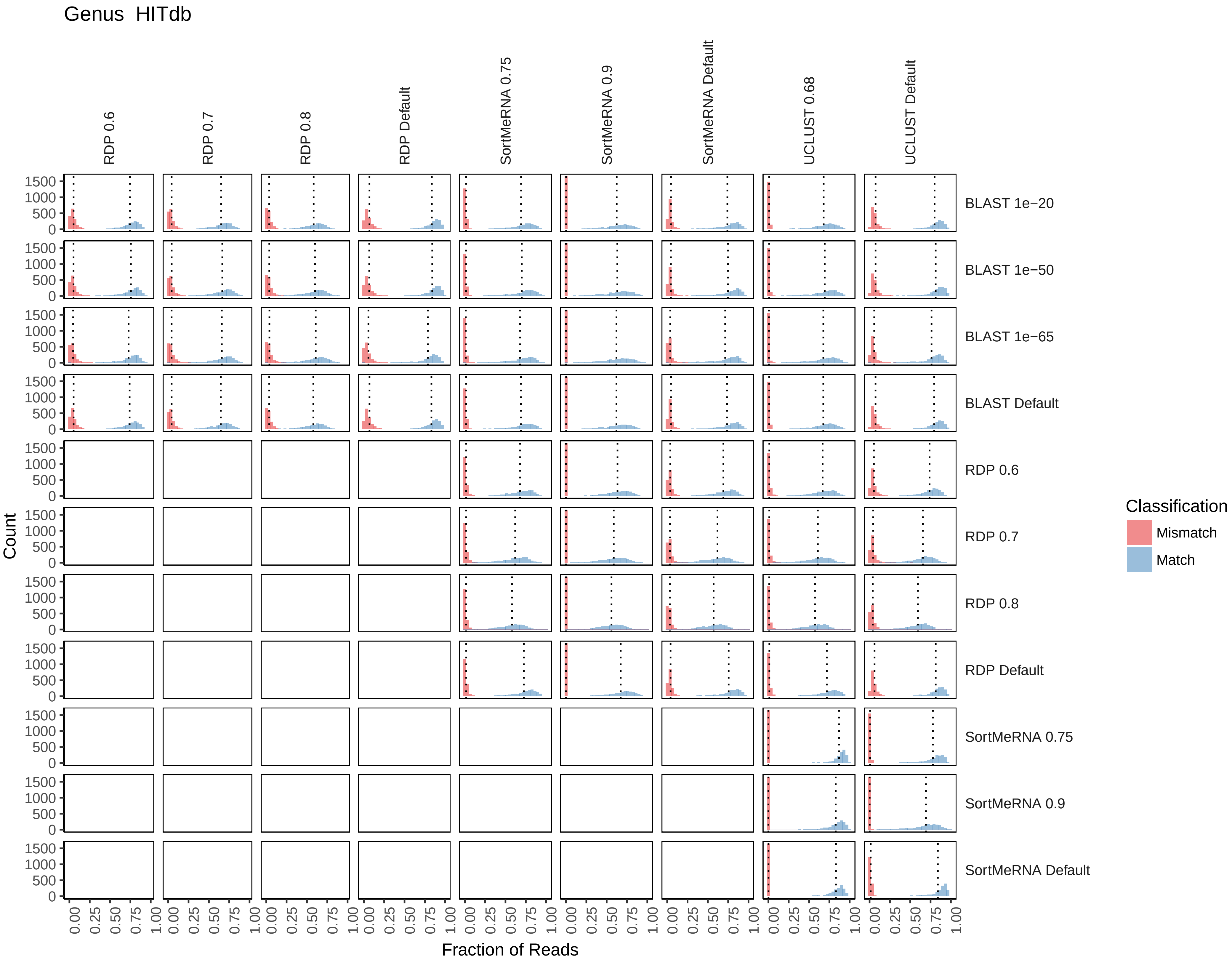

Genus HITdb
RDP 0.6
RDP 0.7
RDP 0.8
RDP Default
SortMeRNA 0.75
SortMeRNA 0.9
SortMeRNA Default
UCLUST 0.68
UCLUST Default
BLAST 1e−20
BLAST 1e−50
BLAST 1e−65
BLAST Default
RDP 0.6
RDP 0.7
RDP 0.8
RDP Default
SortMeRNA 0.75
SortMeRNA 0.9
SortMeRNA Default
Count
Fraction of Reads
0
500
1000
1500
0.00
0.25
0.50
0.75
1.00
Classification
Mismatch
Match

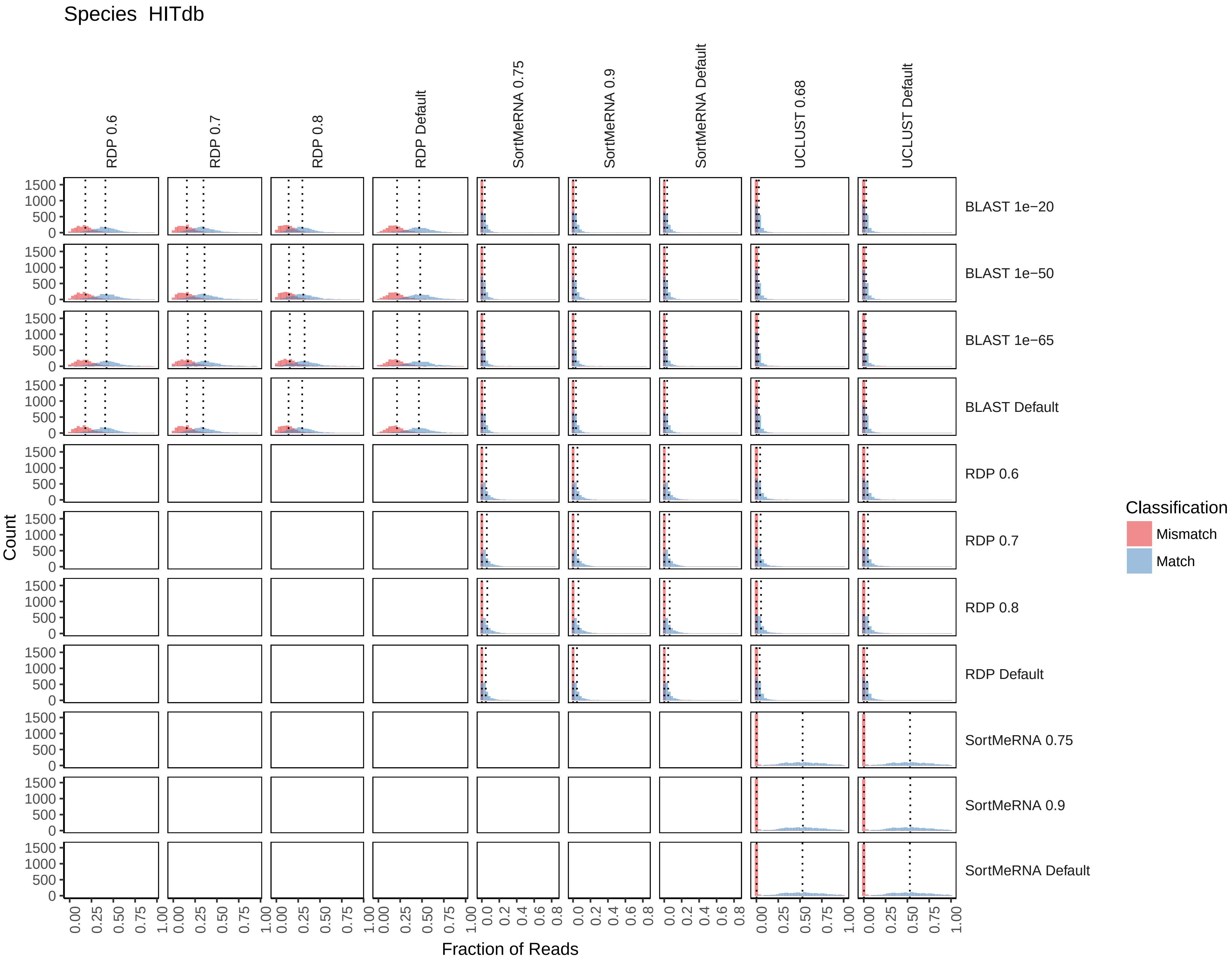

Species HITdb
RDP 0.6
RDP 0.7
RDP 0.8
RDP Default
SortMeRNA 0.75
SortMeRNA 0.9
SortMeRNA Default
UCLUST 0.68
UCLUST Default
BLAST 1e−20
BLAST 1e−50
BLAST 1e−65
BLAST Default
RDP 0.6
RDP 0.7
RDP 0.8
RDP Default
SortMeRNA 0.75
SortMeRNA 0.9
SortMeRNA Default
Count
Fraction of Reads
Classification
Mismatch
Match

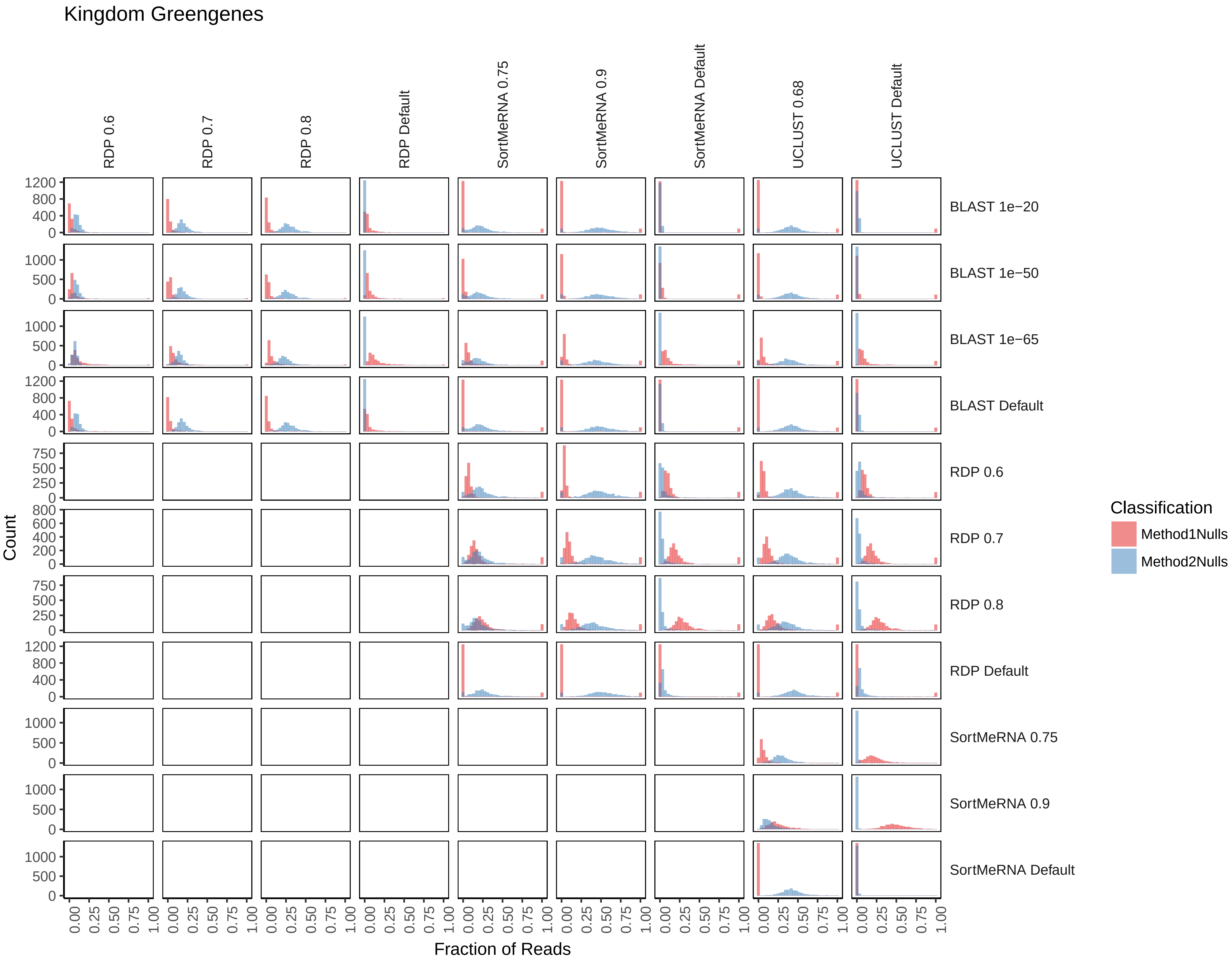

Kingdom Greengenes
RDP 0.6
RDP 0.7
RDP 0.8
RDP Default
SortMeRNA 0.75
SortMeRNA 0.9
SortMeRNA Default
UCLUST 0.68
UCLUST Default
BLAST 1e−20
BLAST 1e−50
BLAST 1e−65
BLAST Default
RDP 0.6
RDP 0.7
RDP 0.8
RDP Default
SortMeRNA 0.75
SortMeRNA 0.9
SortMeRNA Default
Count
Fraction of Reads
0.00 0.25 0.50 0.75 1.00
Classification
Method1Nulls
Method2Nulls

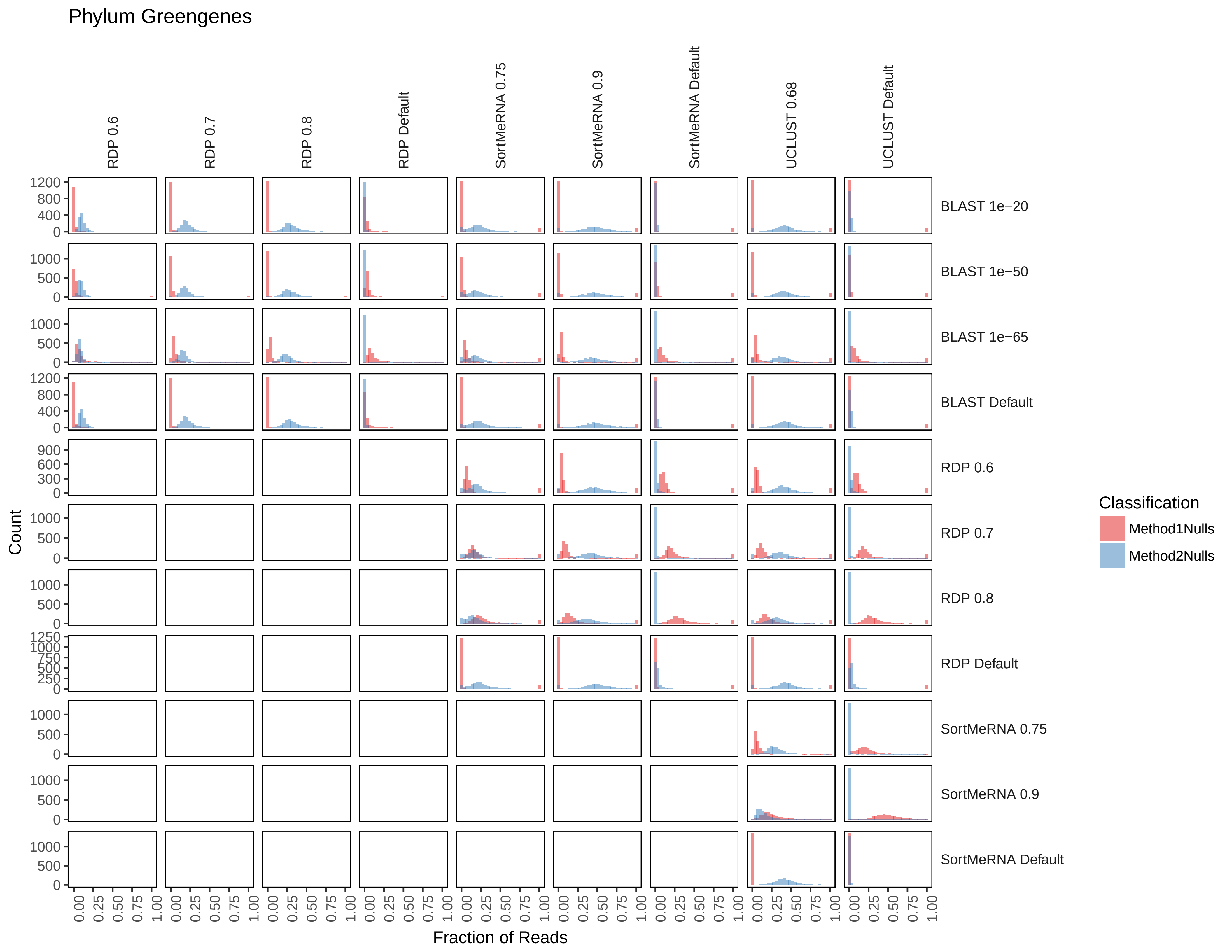
Phylum Greengenes
RDP 0.6
RDP 0.7
RDP 0.8
RDP Default
SortMeRNA 0.75
SortMeRNA 0.9
SortMeRNA Default
UCLUST 0.68
UCLUST Default
BLAST 1e−20
BLAST 1e−50
BLAST 1e−65
BLAST Default
RDP 0.6
RDP 0.7
RDP 0.8
RDP Default
SortMeRNA 0.75
SortMeRNA 0.9
SortMeRNA Default
Count
Fraction of Reads
Classification
Method1Nulls
Method2Nulls

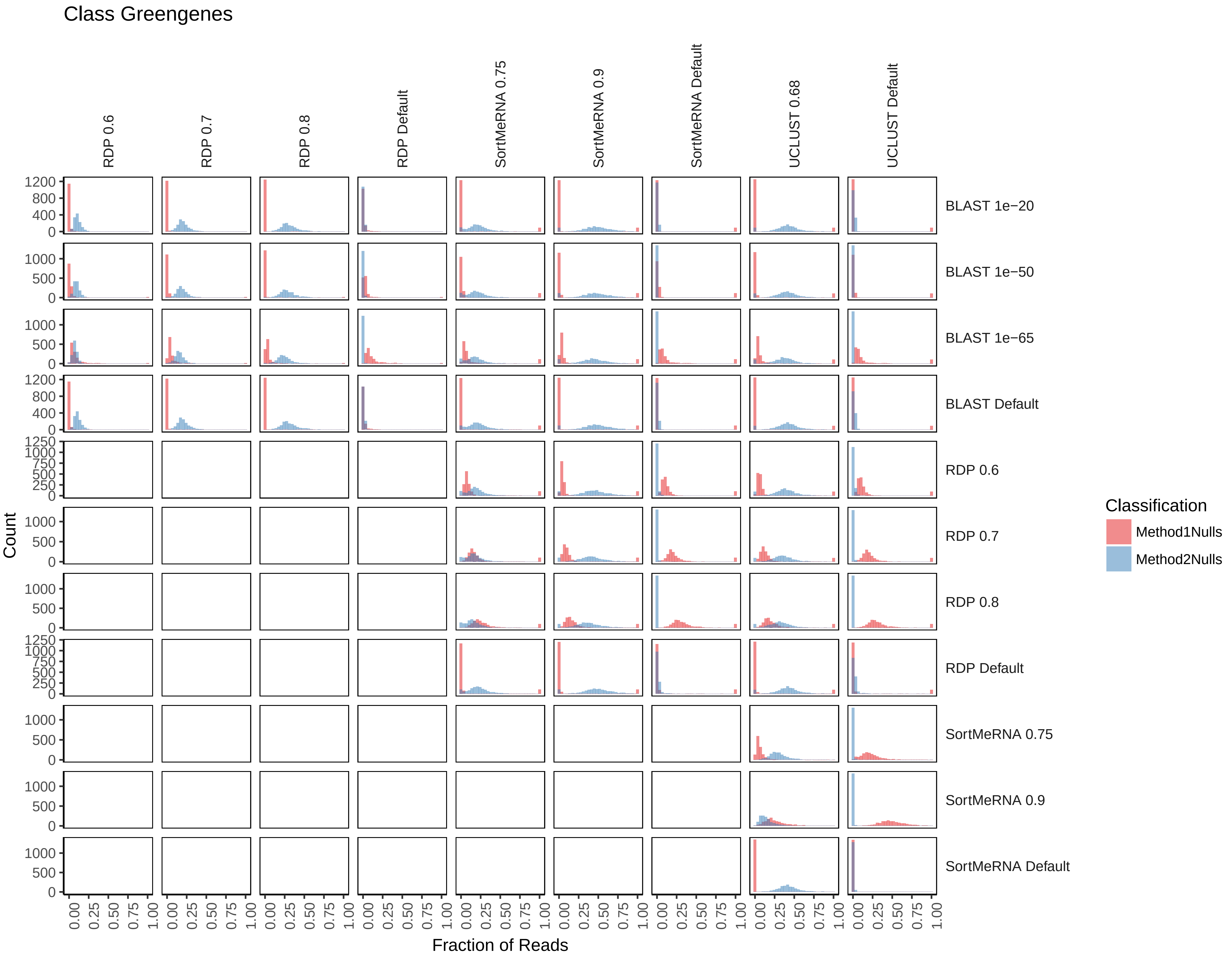
Class Greengenes
RDP 0.6
RDP 0.7
RDP 0.8
RDP Default
SortMeRNA 0.75
SortMeRNA 0.9
SortMeRNA Default
UCLUST 0.68
UCLUST Default
BLAST 1e−20
BLAST 1e−50
BLAST 1e−65
BLAST Default
RDP 0.6
RDP 0.7
RDP 0.8
RDP Default
SortMeRNA 0.75
SortMeRNA 0.9
SortMeRNA Default
Count
Fraction of Reads
Classification
Method1Nulls
Method2Nulls

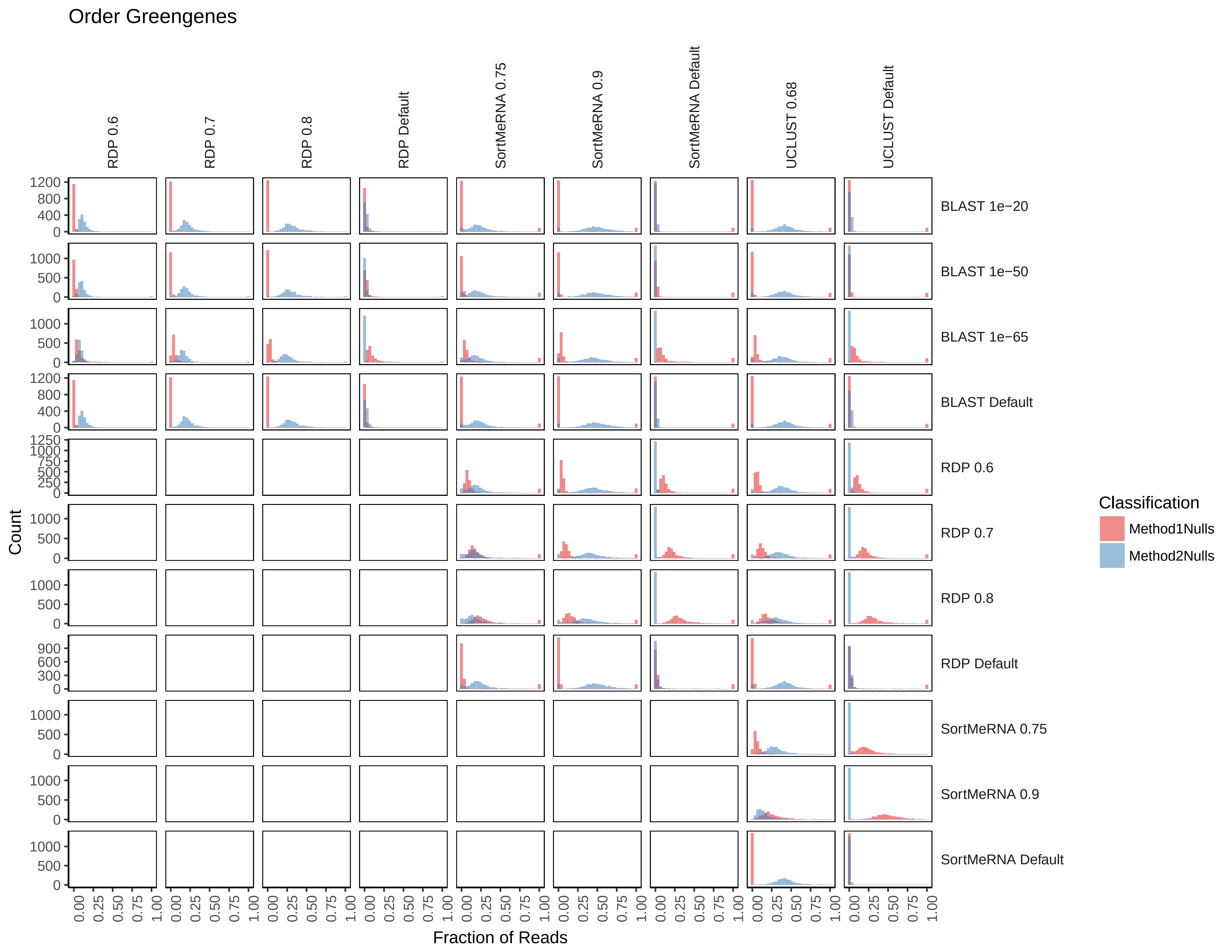
Order Greengenes
RDP 0.6
RDP 0.7
RDP 0.8
RDP Default
SortMeRNA 0.75
SortMeRNA 0.9
SortMeRNA Default
UCLUST 0.68
UCLUST Default
BLAST 1e−20
BLAST 1e−50
BLAST 1e−65
BLAST Default
RDP 0.6
RDP 0.7
RDP 0.8
RDP Default
SortMeRNA 0.75
SortMeRNA 0.9
SortMeRNA Default
Count
Fraction of Reads
0.00 0.25 0.50 0.75 1.00
Classification
Method1Nulls
Method2Nulls

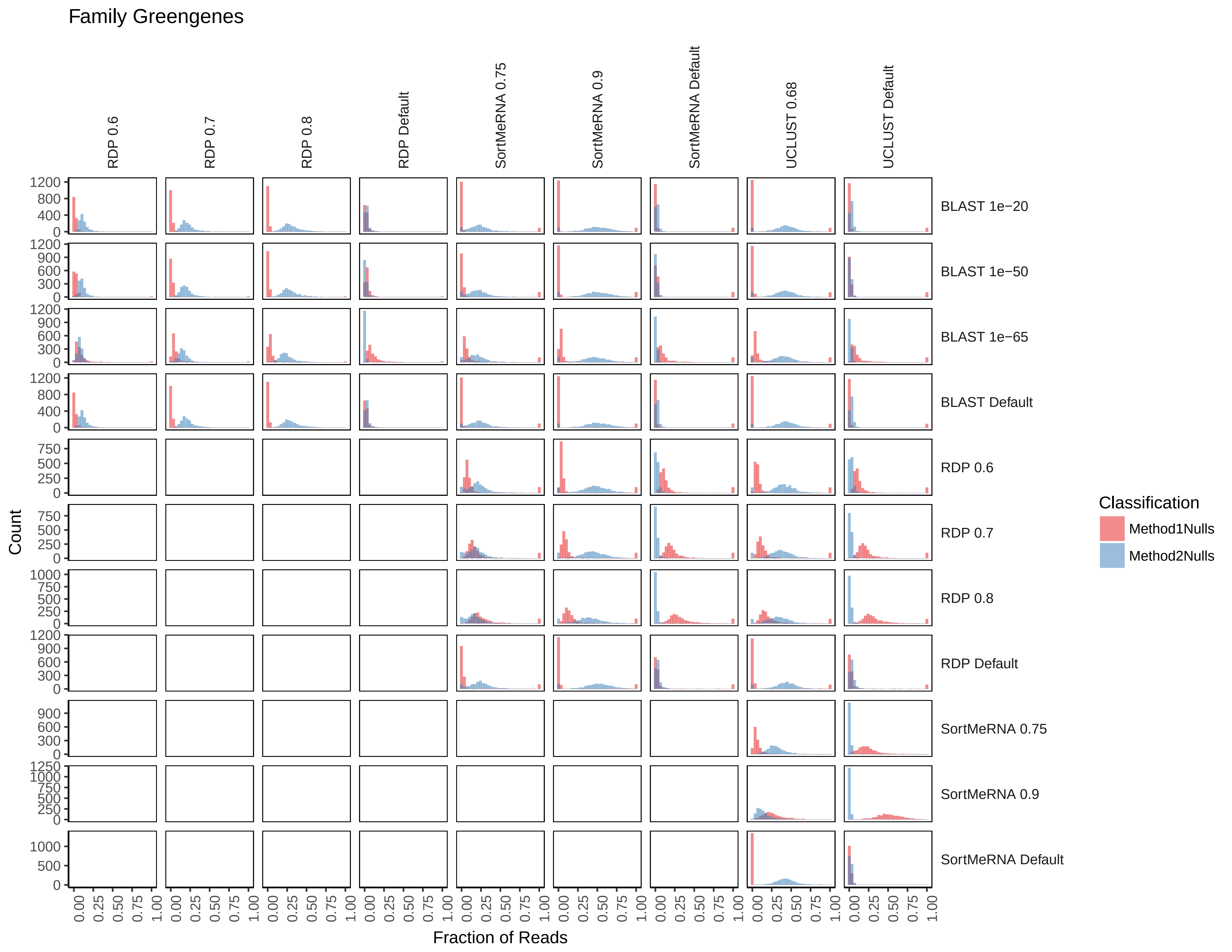
Family Greengenes
RDP 0.6
RDP 0.7
RDP 0.8
RDP Default
SortMeRNA 0.75
SortMeRNA 0.9
SortMeRNA Default
UCLUST 0.68
UCLUST Default
BLAST 1e−20
BLAST 1e−50
BLAST 1e−65
BLAST Default
RDP 0.6
RDP 0.7
RDP 0.8
RDP Default
SortMeRNA 0.75
SortMeRNA 0.9
SortMeRNA Default
Count
Fraction of Reads
Classification
Method1Nulls
Method2Nulls

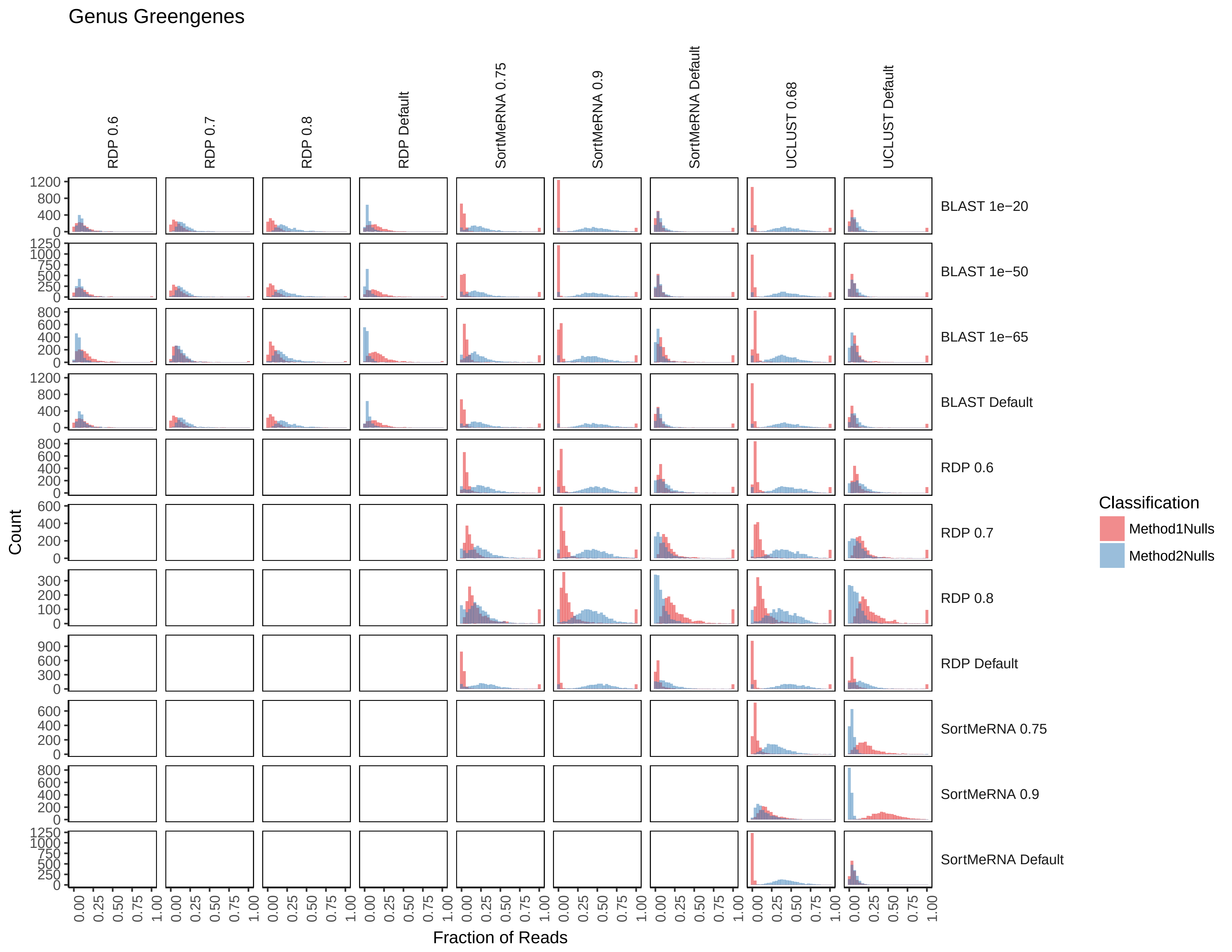

Genus Greengenes
RDP 0.6
RDP 0.7
RDP 0.8
RDP Default
SortMeRNA 0.75
SortMeRNA 0.9
SortMeRNA Default
UCLUST 0.68
UCLUST Default
BLAST 1e−20
BLAST 1e−50
BLAST 1e−65
BLAST Default
RDP 0.6
RDP 0.7
RDP 0.8
RDP Default
SortMeRNA 0.75
SortMeRNA 0.9
SortMeRNA Default
Count
Fraction of Reads
0.00 0.25 0.50 0.75 1.00
Classification
Method1Nulls
Method2Nulls

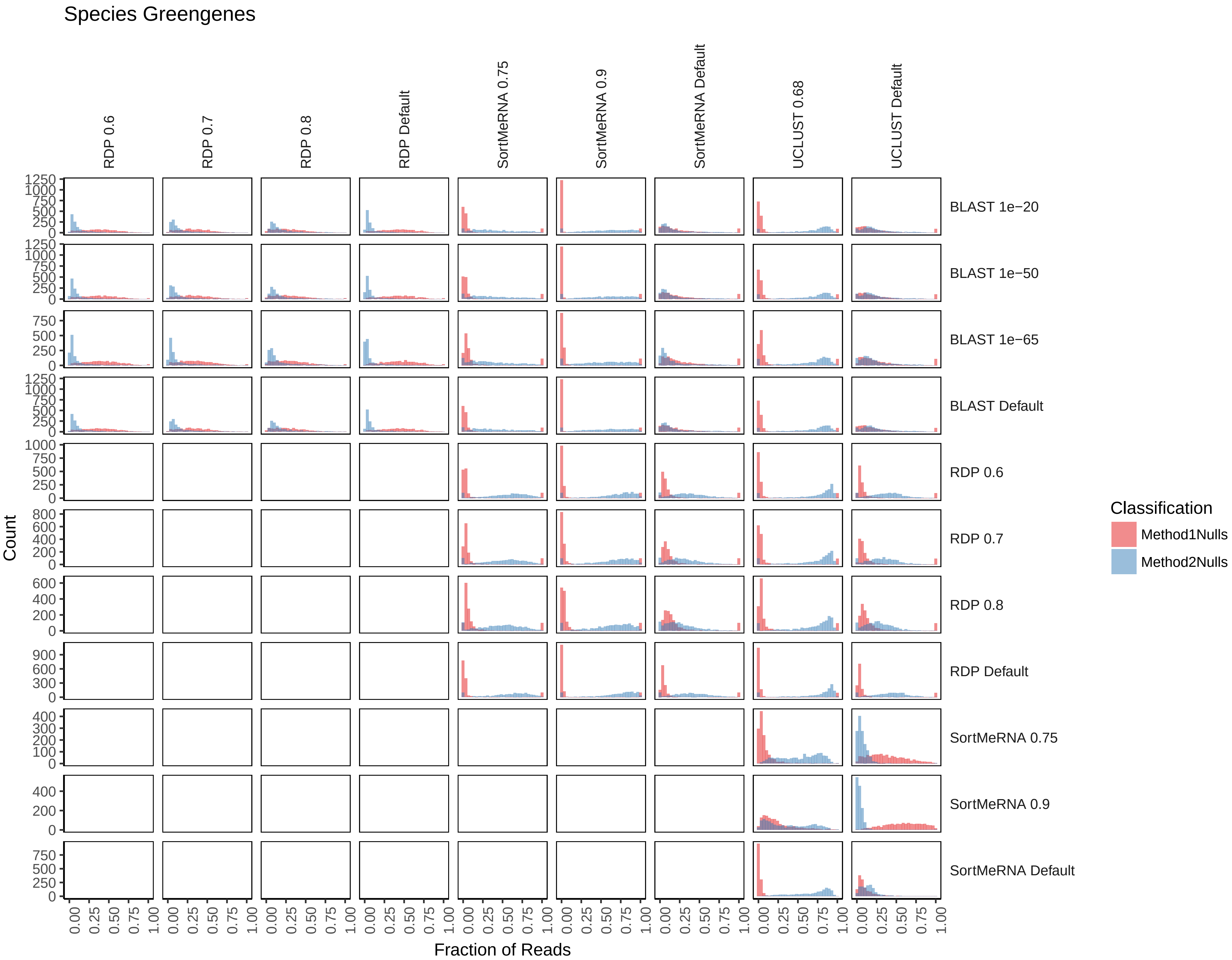

Species Greengenes
RDP 0.6
RDP 0.7
RDP 0.8
RDP Default
SortMeRNA 0.75
SortMeRNA 0.9
SortMeRNA Default
UCLUST 0.68
UCLUST Default
BLAST 1e−20
BLAST 1e−50
BLAST 1e−65
BLAST Default
RDP 0.6
RDP 0.7
RDP 0.8
RDP Default
SortMeRNA 0.75
SortMeRNA 0.9
SortMeRNA Default
Count
Fraction of Reads
Classification
Method1Nulls
Method2Nulls

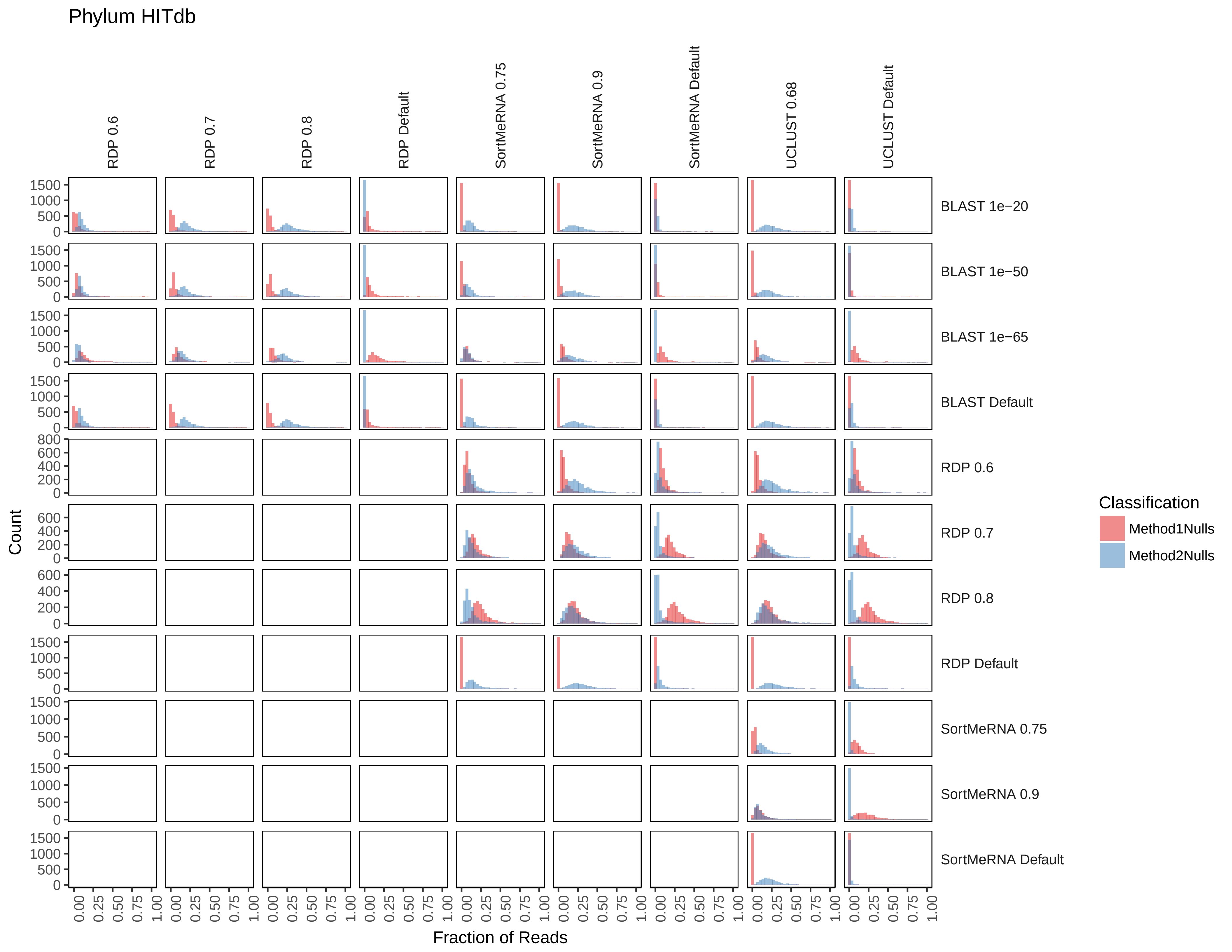

Phylum HITdb
RDP 0.6
RDP 0.7
RDP 0.8
RDP Default
SortMeRNA 0.75
SortMeRNA 0.9
SortMeRNA Default
UCLUST 0.68
UCLUST Default
BLAST 1e−20
BLAST 1e−50
BLAST 1e−65
BLAST Default
RDP 0.6
RDP 0.7
RDP 0.8
RDP Default
SortMeRNA 0.75
SortMeRNA 0.9
SortMeRNA Default
Count
Fraction of Reads
Classification
Method1Nulls
Method2Nulls

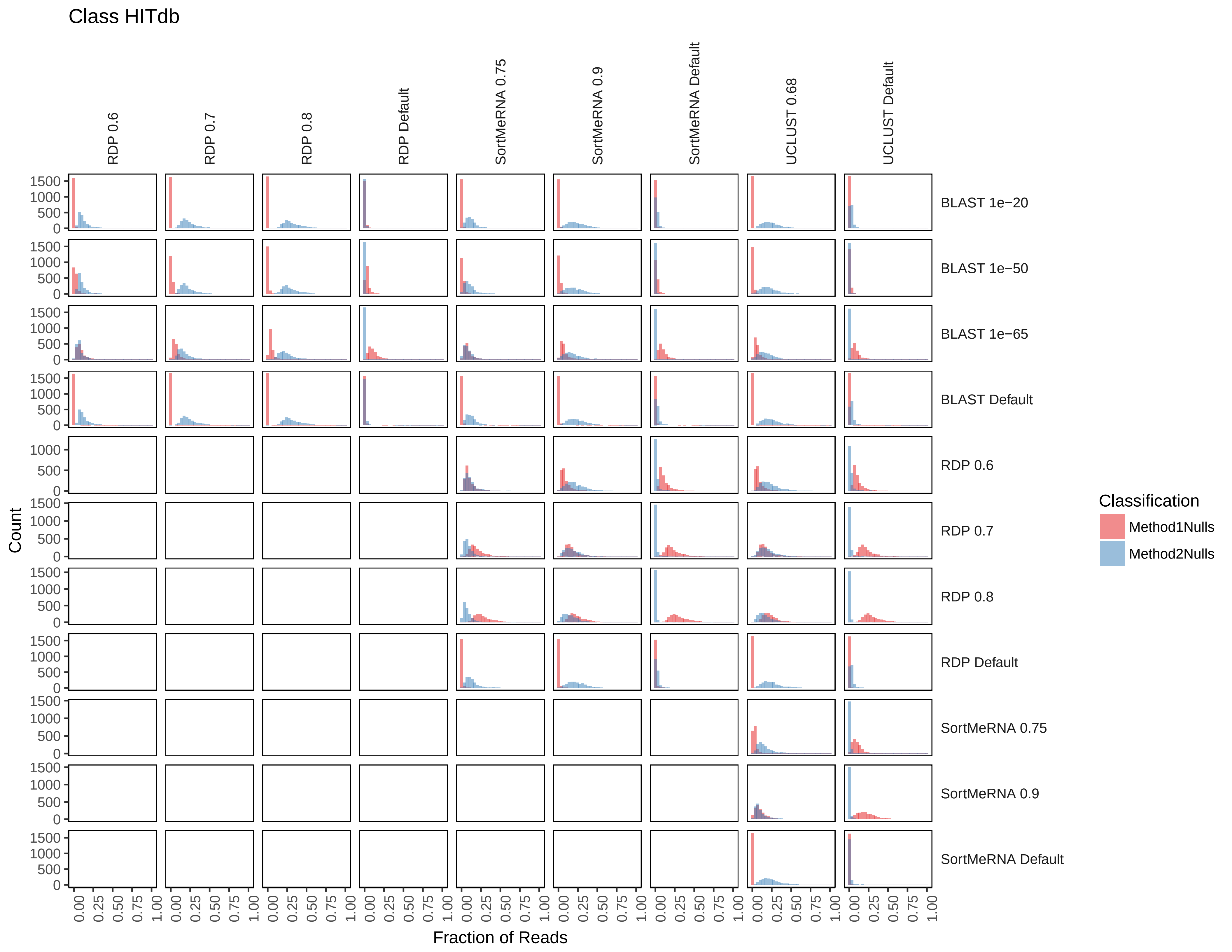

Class HITdb
RDP 0.6
RDP 0.7
RDP 0.8
RDP Default
SortMeRNA 0.75
SortMeRNA 0.9
SortMeRNA Default
UCLUST 0.68
UCLUST Default
BLAST 1e−20
BLAST 1e−50
BLAST 1e−65
BLAST Default
RDP 0.6
RDP 0.7
RDP 0.8
RDP Default
SortMeRNA 0.75
SortMeRNA 0.9
SortMeRNA Default
Count
0
500
1000
1500
Fraction of Reads
0.00
0.25
0.50
0.75
1.00
Classification
Method1Nulls
Method2Nulls

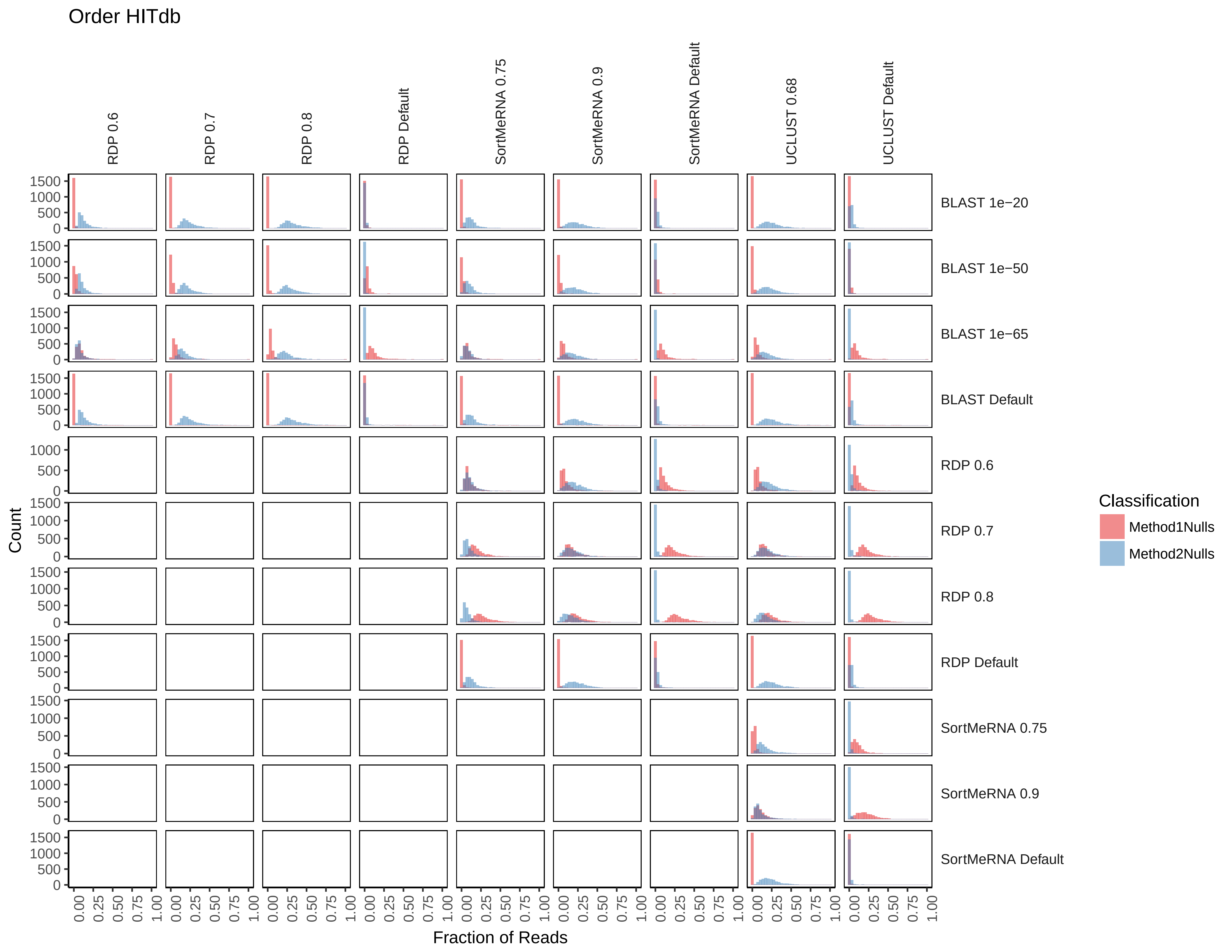

Order HITdb
RDP 0.6
RDP 0.7
RDP 0.8
RDP Default
SortMeRNA 0.75
SortMeRNA 0.9
SortMeRNA Default
UCLUST 0.68
UCLUST Default
BLAST 1e−20
BLAST 1e−50
BLAST 1e−65
BLAST Default
RDP 0.6
RDP 0.7
RDP 0.8
RDP Default
SortMeRNA 0.75
SortMeRNA 0.9
SortMeRNA Default
Count
Fraction of Reads
0.00 0.25 0.50 0.75 1.00
0 500 1000 1500
Classification
Method1Nulls
Method2Nulls

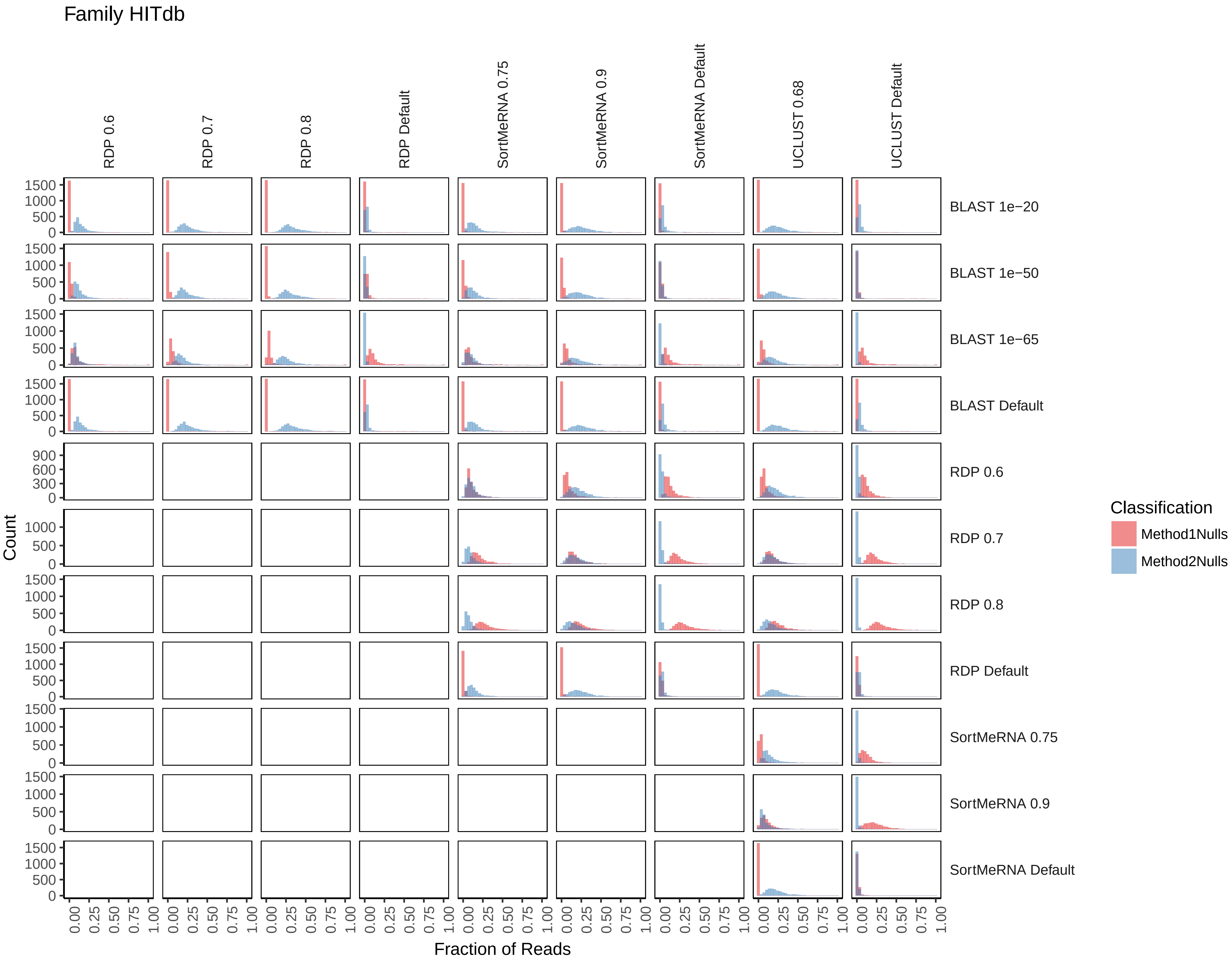

Family HITdb
RDP 0.6
RDP 0.7
RDP 0.8
RDP Default
SortMeRNA 0.75
SortMeRNA 0.9
SortMeRNA Default
UCLUST 0.68
UCLUST Default
BLAST 1e−20
BLAST 1e−50
BLAST 1e−65
BLAST Default
RDP 0.6
RDP 0.7
RDP 0.8
RDP Default
SortMeRNA 0.75
SortMeRNA 0.9
SortMeRNA Default
Count
Fraction of Reads
Classification
Method1Nulls
Method2Nulls

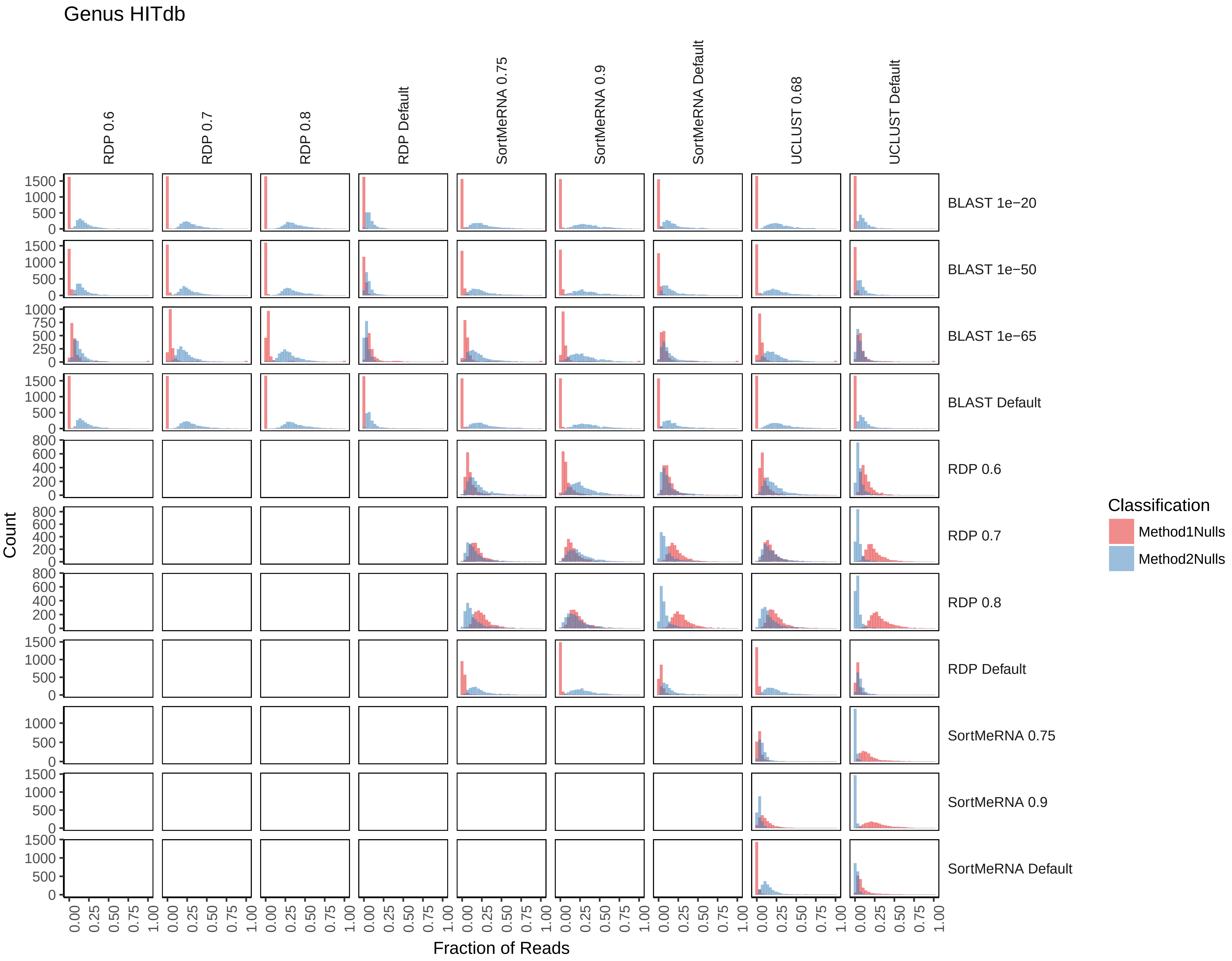
Genus HITdb
RDP 0.6
RDP 0.7
RDP 0.8
RDP Default
SortMeRNA 0.75
SortMeRNA 0.9
SortMeRNA Default
UCLUST 0.68
UCLUST Default
BLAST 1e−20
BLAST 1e−50
BLAST 1e−65
BLAST Default
RDP 0.6
RDP 0.7
RDP 0.8
RDP Default
SortMeRNA 0.75
SortMeRNA 0.9
SortMeRNA Default
Count
Fraction of Reads
0.00 0.25 0.50 0.75 1.00
Classification
Method1Nulls
Method2Nulls

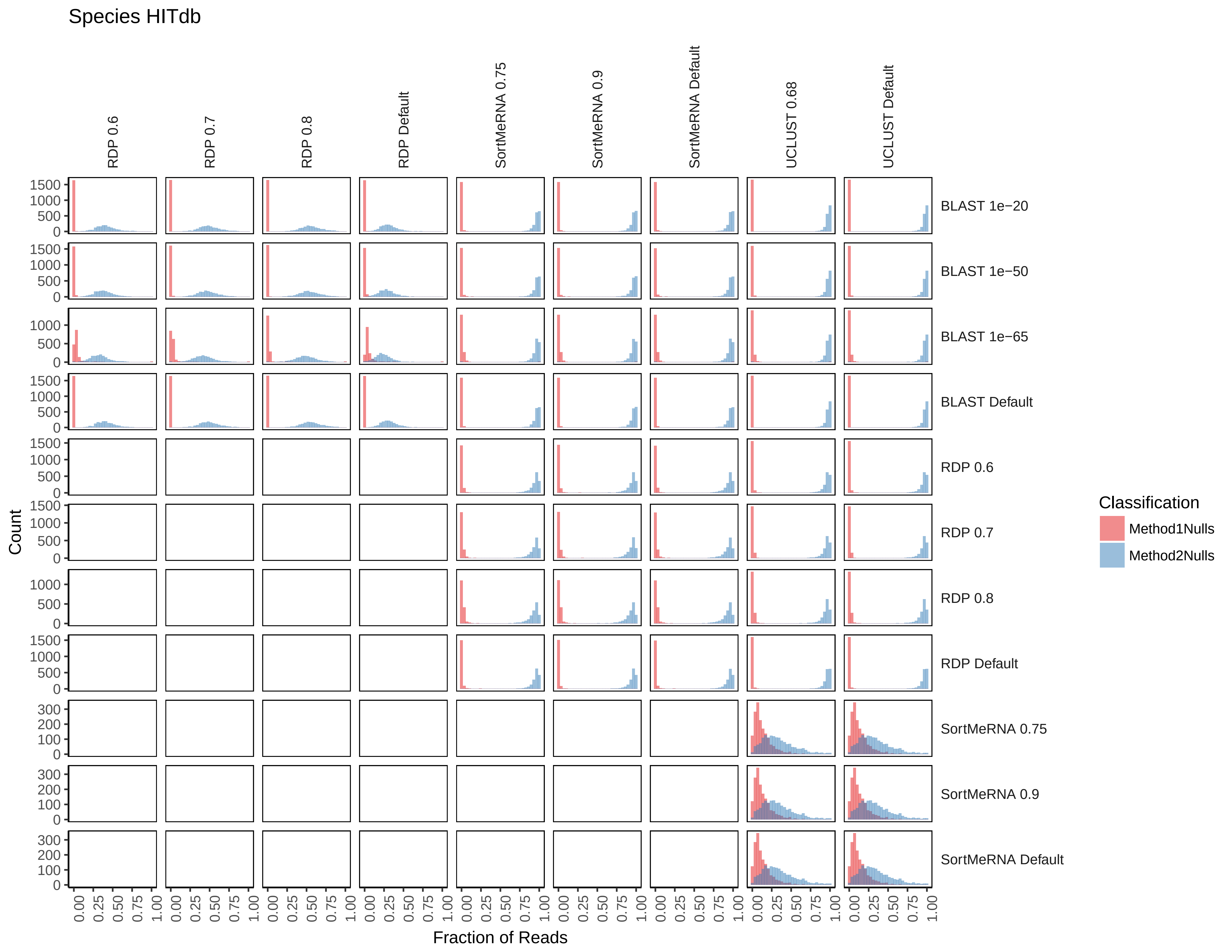

Species HITdb
RDP 0.6
RDP 0.7
RDP 0.8
RDP Default
SortMeRNA 0.75
SortMeRNA 0.9
SortMeRNA Default
UCLUST 0.68
UCLUST Default
BLAST 1e−20
BLAST 1e−50
BLAST 1e−65
BLAST Default
RDP 0.6
RDP 0.7
RDP 0.8
RDP Default
SortMeRNA 0.75
SortMeRNA 0.9
SortMeRNA Default
Count
Fraction of Reads
0.00 0.25 0.50 0.75 1.00
Classification
Method1Nulls
Method2Nulls
